# Targeted metabolomics to quantitatively profile changes in amino acids and phenolics at every step of *amahewu* production from two *Zea Mays* L. maize types (white and yellow)

**DOI:** 10.3389/fnut.2025.1697672

**Published:** 2026-01-12

**Authors:** Sebotse Dolly Kgoale, Abdullahi Adekilekun Jimoh, Fidele Tugizimana, Adrian Abrahams, Oluwafemi Ayodeji Adebo

**Affiliations:** 1 Centre for Innovative Food Research (CIFR), Department of Biotechnology and Food Technology, Faculty of Science, University of Johannesburg, Doornfontein Campus, Johannesburg, South Africa; 2 Research Centre for Plant Metabolomics, Department of Biochemistry, University of Johannesburg, Johannesburg, South Africa; 3 International Research and Development Division, Omnia Group, Ltd., Johannesburg, South Africa; 4 Department of Biotechnology and Food Technology, Faculty of Science, University of Johannesburg, Doornfontein Campus, Johannesburg, South Africa

**Keywords:** metabolomics, metabolites, targeted, phenolics, amino acids

## Abstract

Most existing research on *amahewu* has primarily examined the microbial and physicochemical properties of beverages, leaving the overall metabolite profile insufficiently characterized. Metabolomic analysis enables the monitoring of metabolite shifts throughout fermentation, revealing how microbial processes drive variations in bioactive compounds. Therefore, this study employed targeted metabolomics to investigate dynamic changes in amino acids and phenolic compounds during the production of *amahewu*, a traditional Southern African fermented maize beverage. White and yellow *Zea mays* L. varieties were fermented with malted sorghum inoculum, and samples were analyzed using liquid chromatography–tandem mass spectrometry (LC-MS/MS) with multiple reaction monitoring across four production stages. Chemometric analyses (PCA, OPLS-DA) revealed distinct stage- and maize type-dependent clustering with model validation (*Q*^2^ > 0.5; CV-ANOVA, *p* < 0.05). Across both maize types, seven metabolites discriminated raw from cooked maize, with valine (white maize) and alanine (yellow maize) serving as unique markers. Proline was the most abundant amino acid (>1.0 × 108 ng/g in raw maize), but declined by >80% during fermentation, consistent with microbial utilization. Conversely, threonine and valine increased >3-fold in fermented samples, reflecting proteolysis and microbial synthesis. Apigenin represented >70% of quantified phenolics at all stages, whereas caffeic acid decreased sharply post-cooking (FC = 0.07, *p* = 7.03 × 10^−9^), and avenanthramides emerged *de novo* in fermented samples. Correlation analysis showed strong positive feed-forward associations among amino acids (*r* > 0.8) and feedback-driven phenolic transformations, while pathway enrichment identified isoquinoline alkaloid biosynthesis (impact = 0.41) in white maize and alanine/aspartate/glutamate metabolism (impact = 0.13) in yellow maize as dominant routes. Collectively, this study provides insight into the quantitative changes in metabolic composition at different stages of production, which reflect changes in nutritional, biochemical, and health-promoting properties, as well as microbial communities.

## 1 Introduction

Maize (*Zea mays*) is a monoic annual crop belonging to the family Gramineae and is grown in nearly every part of the world (1). It provides up to 30% of the world's food calories to 4.5 billion people (2, 3). The most used maize types are white and yellow maize, for human consumption (4, 5). These maize types undergo different processes to be edible, including fermentation which can be performed in two ways: the direct fermentation of maize kernels or by first milling the maize to produce a maize meal. Traditional maize fermentation requires minimal ingredients and involves only basic preparation and processing. There is a multitude of diverse maize-fermented beverages, such as *Busaa* (Kenya), *Togwa* (Tanzania), *Ogi* (Nigeria), *Amahewu* (South Africa) *Pozol* (Mexico), and *Chicha* (Brazil), due to numerous food-microbe pairings (3, 6–8). Among these, *amahewu* stands out as one of Southern Africa's most consumed non-alcoholic fermented beverages, with an estimated annual household consumption exceeding 40 million liters across South Africa, Zimbabwe, and neighboring countries (9).

*Amahewu* is valued for its refreshing taste and potential health benefits, which are influenced by its nutritional composition (9, 10). It is used as a refreshing beverage by adults and children in preschool and school-age groups, and as weaning food for infants among low-income rural dwellers (11, 12). The chemistry and metabolite profile of *amahewu* is rich and complex, encompassing a variety of organic acids, vitamins, minerals, amino acids, and bioactive compounds (9). Despite its nutritional and cultural importance, *amahewu* remains largely artisanal, with unstandardized fermentation leading to marked variability in quality, safety, and sensory attributes. The absence of comprehensive biochemical profiling has limited efforts to enhance consistency and nutritional optimization. Thus, metabolomics analysis is urgently needed to elucidate the molecular transformations during fermentation, identify key nutritional and bioactive compounds, and provide a scientific basis for product standardization at both community and industrial scales.

The growth and use of foodomics have kept pace with consumers' growing interest and worry over the ingredients in their food and its safety. This is an emerging interdisciplinary field that integrates advanced omics technologies (such as genomics, transcriptomics, proteomics, metabolomics, and lipidomics) to study food (such as *amahewu*) and its impact on human health and nutrition (13). For this study, we employed targeted metabolomics, a hypothesis-driven approach that focuses on detecting specific metabolites associated with a particular class or metabolic pathway, rather than all components in the sample (14). This helps detect, identify, and monitor changes throughout the production of *amahewu*, as well as related main raw materials and the inoculum used (15). The combination of mass spectrometry and liquid chromatographic separation methods significantly enhances the chemical analysis capabilities of highly complex food samples. This technology enables the physical separation of thousands of metabolites, providing a more comprehensive view of the metabolome (16). The data from the metabolome is multidimensional, intricate, and complex, requiring the application of chemometric approaches to investigate the rich information it contains. Examples of such techniques include orthogonal partial least square discriminant analysis (OPLS-DA) and principal component analysis (PCA), which have been used to give a summary of the dataset, highlighting treatment-related trends, and indicating the metabolites that primarily contribute to differences in treatments, sample sources and other experimental variations (17–20).

As primary and secondary metabolites, amino acids and phenols have many prominent functions. They play pivotal roles in the flavor, nutritional value, and overall quality of food products including *amahewu*. Amino acids are fundamental to protein synthesis, scavenge free radicals and thus have antioxidant properties as well as influence the sensory attributes of the final product, while phenolics are known for their antioxidant properties and contribution to the beverage's taste and color (21). In addition to nutritional and functional interest, amino acids and phenolic acids have been recognized as important biomarkers for foodomics studies (21, 22).

Food processing, such as fermentation produces substantial metabolic changes in food products, highlighting the need to grasp these alterations. Identifying and understanding the metabolites involved is crucial for unraveling the functions and transformations that occur during production. This study aims to profile amino acids and phenolics during *amahewu* production as both white and yellow *Zea mays* L. varieties possess unique features that could influence fermentation dynamics and the resultant *amehewu's* composition. This was carried out by employing targeted metabolomics using LC-MS/MS in combination with multivariate analysis PCA and OPLS-DA to track variations and offer a full profile of amino acids and phenolic content at each stage of *amahewu* production.

## 2 Methods

### 2.1 Sample collection

White maize (*Zea mays* L.) and yellow maize (*Zea mays* L.) grains harvested in 2023 were obtained from the Agricultural Research Council (ARC-LNR Teliersaad breeder seed), Pretoria, South Africa. Sorghum grains (*Sorghum bicolor* (L.) Moench, cultivar NS 5655, 2023 harvest) were sourced from Agricol, Pretoria, South Africa. All samples were transported under dry conditions and stored at room temperature until further use.

### 2.2 Maize preparation

The white and yellow maize grains (4 kg) were weighed using a Mettler Toledo PL202-S scale (Greifensee, Switzerland), washed, and dried overnight at 38 °C in a drying oven (Prolab, Manama, Bahrain). The dried maize grains were milled using a laboratory mill (3600 Perten, Hudding, Sweden) with a 1.5 mm grain size sieve and then passed through a sieve shaker (Analysette 3 Spartan, Fritsch GmbH, Idar-Oberstein, Germany).

### 2.3 Sorghum preparation and malting

Sorghum grains (2 kg) were weighed on a weighing balance (Mettler Toledo PL202-S, Greifensee, Switzerland) and submerged in water for 1 day at room temperature. After a day, the water was drained and the sorghum grains were spread on a tray to germinate at room temperature, with water spraying in between the 48 h of germination. Two to four centimeters of sprouts germinated and were dried at 40 °C overnight in a drying oven (Prolab, Manama, Bahrain). The dried sprouted grains were milled using a laboratory mill (3600 Perten, Hudding, Sweden) with a 1.5 mm grain size and then passed through a sieve shaker (Analysette 3 Spartan, Fritsch GmbH, Idar-Oberstein, Germany).

### 2.4 Preparation of *amahewu*

Approximately 200 g of the milled maize (yellow and white maize) was mixed with 500 mL of autoclaved water in a 1 L autoclaved glass beaker (Sample 1; RWM/RYM). The mixture was cooked on a hot plate at 95 ± 2 °C for 20–30 min with continuous stirring using a sterilized glass rod until a thick porridge was formed (Sample 2; CWM/CYM). After cooking, the porridge was allowed to cool to about 30 °C and transferred into a 1 L Schott bottle. As an inoculum, 30 g of malted sorghum flour was added to the cooled porridge, thoroughly mixed in a 1 L Schott bottle (Sample 3; IWM/IYM), and then incubated at 30 °C (Labcon, Krugersdorp, South Africa) for 26 h to obtain the fermented *amahewu* (WMA/YMA). The malted sorghum used as inoculum was designated as Sample 5 (Sinc).

After each phase and production, samples of raw maize, cooked maize, inoculated paste, and fermented *amahewu* (Table 1) were collected into sterile 50 mL Falcon tubes for subsequent physicochemical and metabolomic analyses. The remaining samples were refrigerated at −80 °C and then freeze-dried using a benchtop freeze dryer (Beijer Electronics HT40; Telstar LyoQuest, Terrassa, Spain). The flours obtained from freeze-drying were stored in Ziplock bags at 4 °C for further analysis.

**Table 1:** Sample details, processing steps and identifications.

**Sample no**	**Processing step**	**Sample ID**
1	Initial (raw white maize)	RWM
2	Cooked white maize	CWM
3	Inoculated maize paste	IWM
4	Fermented (*amahewu*)	WMA
1	Initial (raw yellow maize)	RYM
2	Cooked yellow maize	CYM
3	Inoculated maize paste	IYM
4	Final (*amahewu*)	YMA
5	Inoculum (malted sorghum)	IS

### 2.5 Determination of amino acids and phenolics using liquid chromatography-mass spectrometry

Approximately 2.5 g of each powdered sample was weighed, and 6 mL of 80% methanol was added as an extraction solvent. This corresponds to a solid-to-solvent ratio of approximately 1:2.4 (w/v). The mixture was vortex-mixed for 1 min to ensure uniform dispersion. The solution was ultrasonicated for 30 min (Scientech 704, Labotech, Johannesburg, South Africa) at a temperature of 40 °C, followed by centrifugation for 5 min at 3.000 × g (Eppendorf 5702R, Merck, Johannesburg, South Africa). The supernatant was filtered through a 0.22 μm polyethersulfone membrane filter into vials.

Samples and working solutions of standards were analyzed on an ultra-fast liquid chromatography (UFLC) system, fitted with Shim-pack GIST C18 column (2 μm; 100 × 2.1 mm l.D) (Shimadzu, Kyoto, Japan), housed in an oven with thermostat set at 40 °C. Chromatographic separation was performed using gradient elution, utilizing eluent A (MilliQ water with 0.1% formic acid) and eluent B (methanol with 0.1% formic acid) (Romil Chemistry, UK) at a constant flow rate of 0.2 mL min^−1^. The gradient elution conditions used were as follows: 1 min 98% A, 2% B; 1–3 min 95% A, 5% B; 2 min 90% A 10% B; 2 min 50% A, 50% B, then changed to the initial conditions. The total chromatographic run time was 10 min, and the injection volume was 2 μL for phenolics and 3 μL for amino acids. The MS conditions were as follows: nitrogen was supplied as the drying gas at a flow rate of 10 L min^−1^ and as the nebulizing gas at 3 L min^−1^; the heating gas flow was maintained at 10 L min^−1^. The interface temperature and voltage were set at 300 °C and 4 kV, respectively, while the DL temperature was 250 °C, and the heat block temperature was set to 400 °C. Three biological replicates were prepared for each sample, and each biological replicate was analyzed in triplicate on the LC-MS system.

### 2.6 Data analysis and interpretation

Data generated from LC-MRM-MS (liquid chromatography-mass spectrometry with multiple reaction monitoring) analyses were processed using LabSolution Quant BrowserTM (Shimadzu, Kyoto, Japan), where the concentrations of the unknown samples were extrapolated from the respective calibration curves. From obtaining the extrapolated concentrations expressed in parts per billion (ppb) (for amino acids) and parts per million (ppm) (for phenolics), the final concentrations were calculated and then converted to ng/g, respectively. The generated data was first imported into the SIMCA software, version 14.1 (Umetrics, Umea, Sweden) for chemometrics/multivariate statistical analysis. The imported data was scaled to unit variance prior to PCA and OPLS-DA.

To perform supervised and unsupervised multivariate statistical analysis, PCA was performed to visualize the datasets firsthand and detect outliers, while OPLS-DA was used to assess the methodology's ability to discriminate between production steps, maize type and inoculum. These models were validated after being built using 7-fold cross-validation. For PCA, the cumulative *R*^2^ (explained variation) and *Q*^2^ (predicted variation) of the models used were higher than 0.5. Furthermore, for OPLS-DA models, the analysis of variance testing of cross-validated predictive residuals (CV-ANOVA), *p*-values were below 0.05 (23). All curves showed excellent linearity (*R*^2^ = 0.991–0.999).

The generated concentration data matrix was also uploaded into MetaboAnalyst 5.0 (http://www.metaboanalyst.ca) for metabolite-metabolite correlation analysis (MMCA) using Pearson's correlation function; and for pathways analysis using metabolomics pathway analysis (MetPA) tool, a part of MetaboAnalyst 5.0. Since many pathways are tested at the same time, both Holm-Bonferroni and false discovery rate (FDR) procedures were used to adjust for the statistical *p*-values from enrichment analysis.

## 3 Results and discussion

### 3.1 Multivariate data analysis of amino acids and phenolics profiles from white and yellow maize-produced *amahewu* inoculated with malted sorghum.

**Figure 1:**
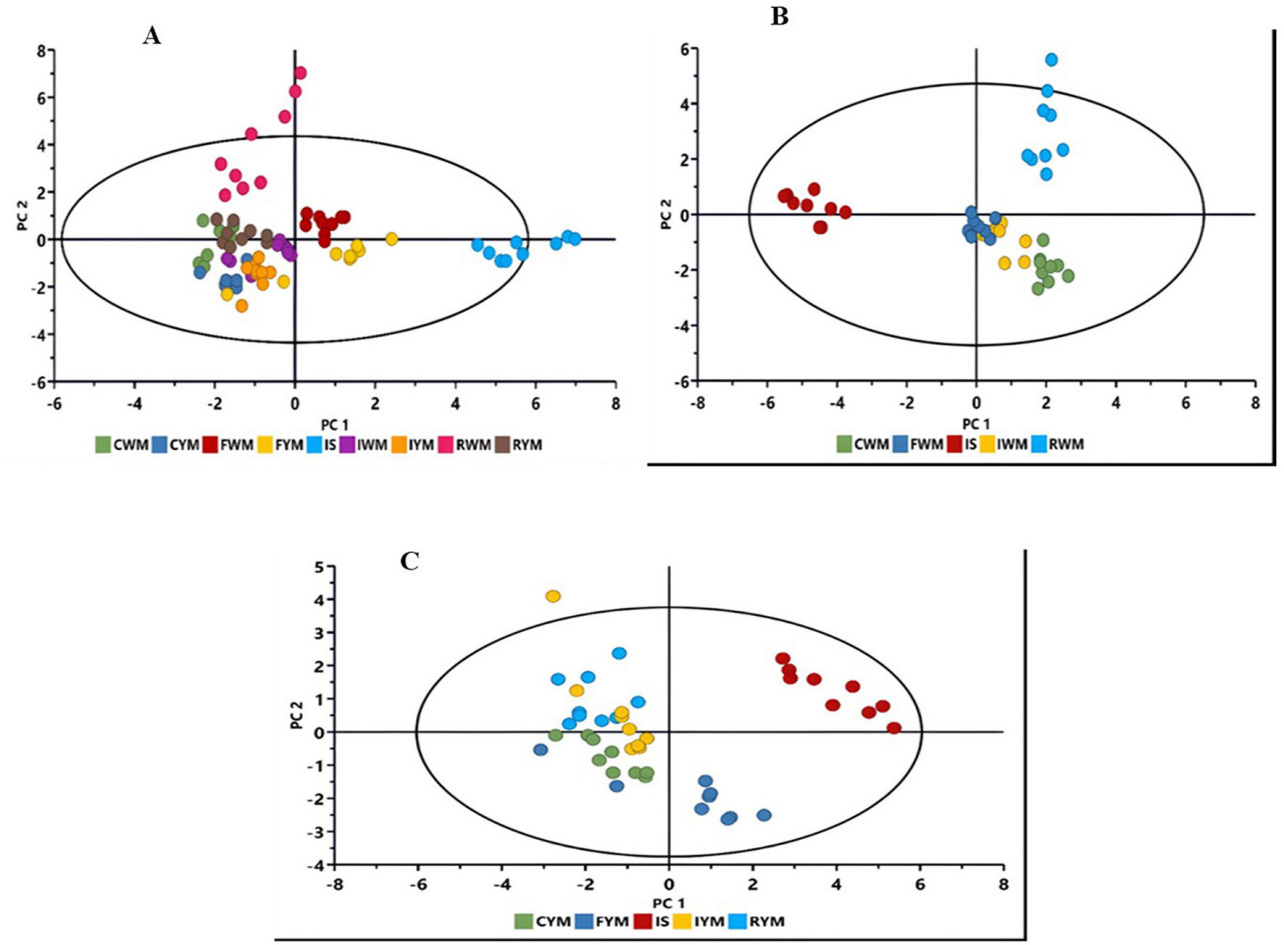
Principal component analysis (PCA) plots displaying the sample clusters that were investigated. **(A)** PCA score plot of both yellow and white maize (PC1; 23.8% vs. PC2; 4.7%). **(B)** PCA score plot of white maize (PC1; 33.4% vs. PC2; 18.6%) (**C**) PCA score plot of yellow maize (PC1; 33.8% vs. PC2; 18.6%). The PCA scores represent the production steps of *amahewu* using white and yellow maize with malted sorghum as an inoculum. CWM, cooked white maize; FWM, fermented white maize; IWM, inoculated white maize; RWM, raw white maize; CYM, cooked yellow maize; FYM, fermented yellow maize; IYM, inoculated yellow maize; RYM, raw yellow maize; IS, inoculated sorghum.

As mentioned in the experimental section, chemometrics modeling was applied to reduce data complexity, enhance interpretation, and identify key metabolic profiles that define the production of *amahewu*. The computed PCA model revealed sample groupings and natural trends related to maize type (yellow vs. white), treatment (fermented vs. non-fermented), and different production stages (Figure 1). Figure 1A shows a clear separation of sample groups based on their metabolite profiles, with IS, CYM, and RYM forming distinct clusters away from the central overlapping groups (CWM, FWM, FYM, IWM, IYM, and RWM). The tight clustering of IS indicates high within-group similarity, while the wide separation of CYM along principal component 1 (PC1) and RYM along principal component 2 (PC2) suggests that these groups possess unique biochemical compositions compared to the others. In contrast, the central overlap of several groups reflects shared metabolic features and less pronounced differentiation.

In the score plots indicating white maize production, a clear separation of IS and FWM from the other groups was observed, with IS positioned at the top and FWM at the lower end, exhibiting minimal overlaps. RWM is distinctly separated at the top, while CWM and IWM clustered closely together at the lower region (Figure 1B). In the score plots indicating yellow maize production, RYM, CYM, and IYM are closely clustered to each other (Figure 1C). The separation and clustering patterns highlight the extent of metabolic alterations occurring during the processing of *amahewu*, particularly the influence of the inoculum on the metabolite composition of the final products.

**Figure 2:**
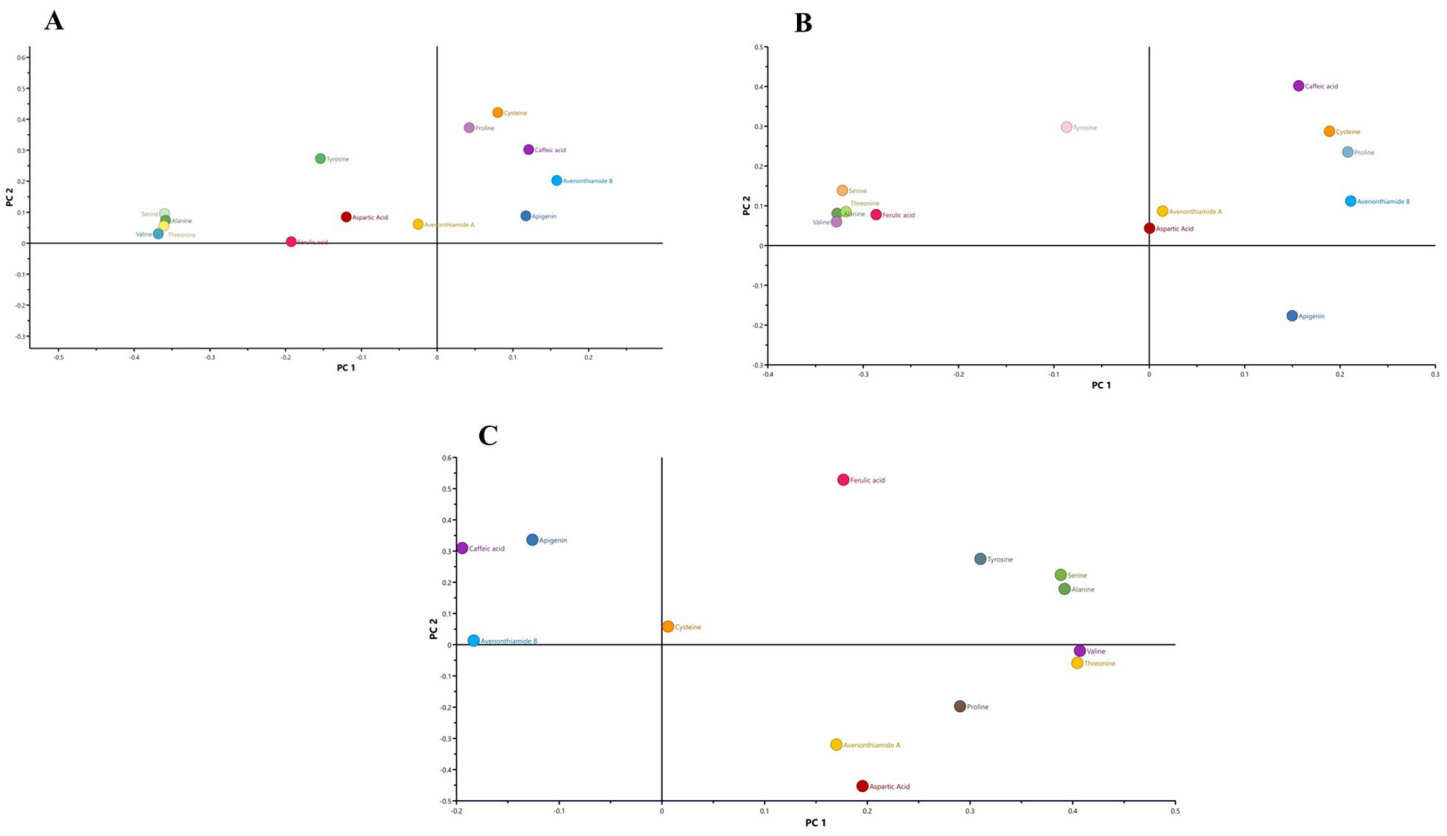
Principal component analysis (PCA) loading plots displaying the discriminant metabolites **(A)** yellow and white maize (PC1; 23.8% vs. PC2; 4.7%), (**B**) white maize (PC1; 33.4% vs. PC2; 18.6%), (**C**) yellow maize (PC1; 33.8% vs. PC2; 18.6%).

The PCA loading plots revealed clear metabolic differentiation across the production stages of *amahewu* (Figure 2). In Figure 2A, samples representing the final product and inoculum were separated along the positive axis of PC1, whereas the raw, cooked, and inoculated pastes clustered negatively, indicating progressive biochemical changes associated with fermentation. For white maize (Figure 2B), the RWM and IWM stages clustered with caffeic acid, apigenin, and ferulic acid, compounds typically found in unfermented maize. The final product (FWM) correlated with valine, threonine, and proline, suggesting increased amino acid synthesis during fermentation. Similarly, in yellow maize (Figure 2C), RYM and IYM samples were closely associated with apigenin and caffeic acid, while the FYM aligned with amino acids such as valine, threonine, and proline. The inoculum occupied a central position, linked to ferulic acid and serine, reflecting its intermediate enzymatic role in initiating the fermentation process.

The unsupervised chemometric analysis therefore underscores the transformative effect of fermentation on the metabolome of cooked maize and the baseline metabolic differences between raw maize and malted sorghum. These differences in grouping reflect underlying metabolic profiles shaped by carbohydrate availability, enzymatic activity, and secondary metabolite release. Raw maize is rich in starch, which is structurally resistant to enzymatic hydrolysis due to its compact granule structure and higher amylose-to-amylopectin ratio (24). This makes raw maize less directly fermentable, as microbial metabolism requires additional enzymatic activity to liberate fermentable sugars (25). Conversely, malted sorghum exhibits elevated endogenous enzymatic activity, particularly α-amylases and β-glucanases, which are activated and hydrolyze starches into simpler sugars, enhancing microbial accessibility and fermentation processes (26, 27). The close clustering of intermediate maize samples (CWM, IWM, CYM, IYM) suggests gradual yet coordinated biochemical transitions, where starch hydrolysis, protein breakdown, and phenolic release occur incrementally.

### 3.2 Discriminant analysis of metabolites in white maize vs. yellow maize *amahewu* samples

Orthogonal partial least square discriminant analysis was employed to further interpret differential metabolite profiles, separating multivariate relationships into predictive and orthogonal variation and identifying the metabolic features driving groups discrimination. The computed and validated models showed strong classification with no overfitting, as confirmed by cross-validation and permutation testing (*n* = 50). Discriminant biomarkers (unique Rt and *m/z* values) were identified from S-plots and confirmed with VIP scores, which rank variables by contribution and stability (Supplementary Figures S1–18). Thus, statistically significant features contributing to class separation are present in Table 2. The resultant OPLS-DA model comparisons across white and yellow maize, as well as production steps (RWM vs. RYM, CWM vs. CYM, IWM vs. IYM, and FWM vs. FYM), showed good sample classification, suggesting that the two groups have differential metabolite profiles.

**Table 2:** Significant metabolites contributing to the differences at different stages of production.

**Metabolites**	**Rt (min)**	***m/z***	**Class**	**RWM vs. CWM**	**CWM vs. IWM**	**IWM vs. FWM**	**IWM vs. IS**	**RYM vs. CYM**	**CYM vs. IYM**	**IYM vs. FYM**	**IYM vs. IS**
**Amino acids**											
Tyrosine	3.772	136.1	AA	FC: 0.22, *p*: 3.38 × 10^−7^	FC: 3.99, *p*: 1.98 × 10^−8^	FC: 3.99, *p*: 1.98 × 10^−8^	FC: 0.97, *p*: 0.84228	FC: 0.81, *p*: 0.000678	FC: 1.28, *p*: 4.35 × 10^−6^	FC: 0.89, *p*: 0.254303	FC: 1.84, *p*: 0.000747
Serine	1.341	106.2	AA	FC: 0.37, *p*: 0.006145	FC: 1.39, *p*: 0.117021	FC: 1.23, *p*: 0.078313	FC: 6.48, *p*: 7.49 × 10^−12^	FC: 0.61, *p*: 0.00036	FC: 1.28, *p*: 0.025794	FC: 1.37, *p*: 0.0789452	FC: 5.00, *p*: 7.98 × 10^−12^
Cysteine	1.317	241.2	AA	FC: 0.53, *p*: 0.0207	FC: 0.16, *p*: 1.98 × 10^−6^	FC: 3.17, *p*: 2.14 × 10^−12^	FC: 1.10, *p*: 0.465477	FC: 0.53, *p*: 0.002854	FC: 1.40, *p*: 0.054318	FC: 0.86, *p*: 0.346465	FC: 0.79, *p*: 0.054166
Threonine	1.409	120.2	AA	FC: 0.49, *p*: 0.008673	FC: 1.96, *p*: 0.000284	FC: 2.14, *p*: 2.20 × 10^−8^	FC: 3.06, *p*: 7.36 × 10^−9^	FC: 0.74, *p*: 0.008717	FC: 3.89, *p*: 3.38 × 10^−5^	FC: 3.89, *p*: 3.38 × 10	FC: 5.70, *p*: 2.70 × 10^−10^
Valine	1.73	118.2	AA	FC: 0.46, *p*: 0.034181	FC: 2.99, *p*: 8.20 × 10^−5^	FC: 1.64, *p*: 0.004158	FC: 2.99, *p*: 7.90 × 10^−7^	FC: 4.17, *p*: 4.01 × 10^−7^	FC: 3.88, *p*: 0.000428	FC: 3.87, *p*: 0.000428365	FC: 6.84, *p*: 9.17 × 10^−9^
Alanine	1.406	90.2	AA	FC: 0.85, *p*: 0.401191	FC: 0.79, *p*: 0.102804	FC: 1.33, *p*: 0.001631	FC: 5.69, *p*: 2.45 × 10^−10^	FC: 1.81, *p*: 8.77 × 10^−5^	FC: 5.95, *p*: 3.68 × 10^−10^	FC: 1.21, *p*: 0.346495	FC: 5.95, *p*: 3.68 × 10^−10^
Aspartic acid	1.41	134.05	AA	FC: 0.94, *p*: 0.771817	FC: 0.54, *p*: 6.48 × 10^−4^	FC: 3.72, *p*: 3.23 × 10^−12^	FC: 1.75, *p*: 2.35 × 10^−5^	FC: 1.03, *p*: 0.753906	FC: 0.98, *p*: 0.910194	FC: 2.36, *p*: 0.000537	FC: 1.30, *p*: 0.00577721
Proline	1.503	116.2	AA	FC: 0.70, *p*: 0.072321	FC: 0.91, *p*: 0.507674	FC: 0.82, *p*: 0.079015	FC: 0.53, *p*: 0.00013	FC: 1.03, *p*: 0.740362	FC: 1.04, *p*: 0.817874	FC: 1.05, *p*: 0.813527	FC: 1.30, *p*: 0.112155
**Phenolic acids**											
Caffeic acid	9.745	181.16	PC	FC: 0.07, *p*: 7.03 × 10^−9^	FC: 3.85, *p*: 0.003419	FC: 0.76, *p*: 0.363308	FC: 0.26, *p*: 0.00388974	FC: 0.45, *p*: 0.003161	FC: 1.23, *p*: 0.759272	FC: 0.10, *p*: 3.76 × 10^−7^	FC: 0.10, *p*: 3.76 × 10^−7^
Ferulic acid	12.418	195.18	PC	FC: 0.80, *p*: 0.533729	FC: 0.48, *p*: 0.062928	FC: 1.59, *p*: 0.023999	FC: 7.96, *p*: 2.54 × 10^−10^	FC: 0.49, *p*: 0.098054	FC: 3.27, *p*: 0.000927	FC: 0.19, *p*: 0.000248648	FC: 1.35, *p*: 0.072352
Apigenin	14.617	239.25	PC	FC: 2.25, *p*: 0.003336	FC: 0.29, *p*: 0.001362	FC: 1.95, *p*: 0.099032	FC: 0.90, *p*: 0.806254	FC: 0.51, *p*: 0.018635	FC: 1.22, *p*: 0.491707	FC: 0.60, *p*: 0.03302	FC: 0.84, *p*: 0.51281
Avenonthiamide A	17.081	300.28	PC	FC: 1.02, *p*: 0.962071	FC: 0.77, *p*: 0.392877	FC: 0.61, *p*: 0.029158	FC: 1.13, *p*: 0.610648	FC: 2.51, *p*: 0.001558	FC: 0.60, *p*: 0.051619	FC: 1.42, *p*: 0.162371	FC: 1.48, *p*: 0.189886
Avenonthiamide B	17.493	330.3	PC	FC: 0.80, *p*: 0.533729	FC: 2.94, *p*: 4.00 × 10^−7^	FC: 2.94, *p*: 4.00 × 10^−7^	FC: 0.49, *p*: 0.610648	FC: 0.82, *p*: 0.602166	FC: 0.52, *p*: 0.006089	FC: 0.61, *p*: 0.017119	FC: 2.82, *p*: 0.0025237

AA, amio acid; FC, fold change; PC, phenolic compound.

Across both white and yellow maize productions, seven metabolites discriminate raw maize from cooked maize paste, with five AAs and two PCs. However, the specific discriminants differed: valine characterized white maize, whereas alanine was unique to yellow maize. Both are alkyl nonpolar amino acids compared to the other three AAs (cysteine, serine, and tyrosine), which are neutral polar. Between cooked and inoculated maize, eight metabolites discriminate in white maize compared to only five in yellow maize, with overlap restricted to three shared metabolites. Distinct differences included cysteine, aspartic acid, threonine, valine, and apigenin in white maize, and serine and ferulic acid in yellow maize. At the final stage, discriminants between inoculated maize and the final product comprised eight metabolites in white maize (five AAs and three PCs) vs. five in yellow maize (three AAs and two PCs). Here, alanine, cysteine, and avenonthiamide A characterized white maize, while apigenin, a phenolic compound, uniquely defined yellow maize.

The significant metabolites in white maize production increased from seven in RWM vs. CWM and CWM vs. IWM to eight in IWM vs. FWM (Table 2). Serine is only present in the first discriminant and the change addition of aspartic acid and avenonthiamide B in the second discriminant. The final stage added alanine, avenonthiamide A, and ferulic acid, whereas cysteine, threonine, and valine were consistently present throughout. In yellow maize, there was a decrease in fluctuations; seven metabolites were present as significant discriminants between RYM vs. CYM, five in CYM vs. IYM and six in IYM vs. FYM (Table 2). Avenonthiamide B and ferulic acid were additional changes in the second discrimination, complementing tyrosine, serine and cysteine that were present in the first discrimination, and the additional changes of aspartic acid, threonine, valine and apigenin in the final discrimination.

The structural characterization of metabolites, supported by OPLS-DA analysis, demonstrated that the metabolic profiles of the groups were considerably different across production stages. Complementary microbial analysis further revealed dynamic shifts in microbial populations during fermentation, indicating that microbial succession plays a central role in shaping metabolite profiles. This is consistent with the bacterial community structure previously reported by Daji, Green (28), which identified a diverse assemblage of genera, including *Paenibacillus, Weissella, Lactococcus, Enterococcus, Bacillus, Leuconostoc, Pseudomonas*, and *Pediococcus*, in optimized white- and yellow-maize mahewu. Their 16S rRNA profiling demonstrated that microbial diversity and fermentation conditions drive the acidification and flavor development of the beverage (28). Besides lactic acid bacteria (LAB), *amahewu* fermentation involves other microbial consortia comprising yeasts, *Bacillus*, and *Enterobacter species*. These organisms contribute to amino-acid metabolism and phenolic biotransformation, collectively driving the biochemical remodeling observed in the metabolomic profiles. The amino acid profile is particularly critical, as it directly reflects the nutritional quality of proteins and follows similar patterns of protein turnover and degradation (29). Notably, white maize production stages exhibited a greater increase in amino acid metabolites compared to yellow maize, suggesting more extensive proteolytic activity and microbial remodeling in white maize.

### 3.3 Quantification analysis of the discriminating metabolites

**Figure 3:**
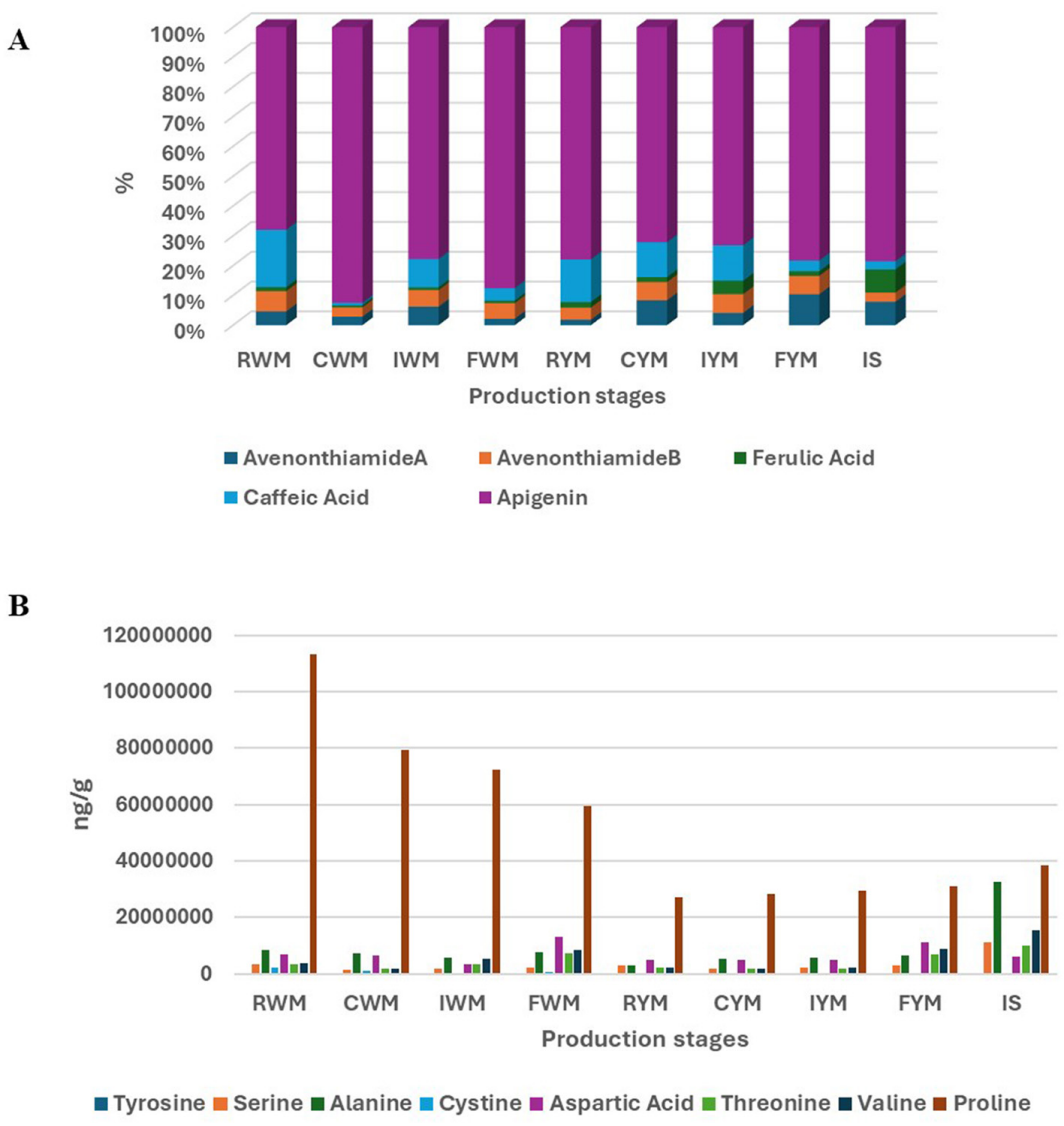
Targeted differential profile during different stages of *amahewu* production (A) Phenolics, (B) Amino acids.

To gain a deeper understanding of the biochemical differences underlying the observed group separations, the discriminating metabolites identified through multivariate analyses were quantitatively assessed (Figure 3). Apigenin was the most abundant compound throughout all stages, consistently representing more than 70% of the total quantified phenolics (Figure 3A). This is expected, as maize naturally contains significant levels of apigenin-C-glycosides such as vitexin and orientin, which can undergo enzymatic hydrolysis during fermentation to release free apigenin (30, 31). The persistence of apigenin across all stages, including after heat treatment and extended fermentation, highlights its stability under thermal and acidic conditions, as well as its resistance to microbial degradation. This stability, coupled with its continuous liberation during fermentation, underscores its importance as a major bioactive contributor in *amahewu*, associated with biological properties (32).

Avenonthiamides commonly referred to as Avenanthramides (Figure 3A) are phenolic amides primarily associated with oats but can also appear in maize through contamination or in sorghum via microbial activity (33). The malting sorghum introduces enzymes and LAB that can hydrolyze bound phenolics from cell walls (34) and conjugate them with amino acids. Consequently, the presence of anthranilic acid or its precursors from protein degradation in maize or sorghum, together with abundant phenolics such as p-coumaric acid and ferulic acid, can promote condensation reactions, ultimately leading to the formation of avenanthramide-like compounds.

Ferulic and caffeic acids, common hydroxycinnamic acids in maize and sorghum, were detected in moderate amounts. Both compounds are typically present in bound forms within the cell wall matrix, and their release depends on thermal softening and enzymatic hydrolysis during fermentation. For instance caffeic acid, a naturally occurring hydroxycinnamic acid widely distributed in cereals, fruits, and vegetables, contributed significantly to the discrimination between raw maize and cooked maize but not between the malted sorghum inoculum and the final products. Its presence at the early stages of processing aligns with reports that cooking enhances the release of bound phenolic acids from cell wall matrices (35). However, its absence in the final fermented products can be attributed to microbial metabolism. LAB and other fermentative microorganisms are known to decarboxylate, reduce, or conjugate caffeic acid into derivatives such as ferulic acid, dihydrocaffeic acid, and their glucosides, effectively reducing its detectable concentration in the end product (14, 36). The contrasting behavior of amino acids and phenolic acids in the discriminant profiles highlights the interplay between proteolysis and phenolic metabolism as central metabolic processes shaping the biochemical fingerprint of fermented maize beverages.

For quantification of the discriminant amino acids, proline exhibited the highest concentrations across all stages, particularly in RWM and CWM, where its levels exceeded 100 million units, making it the predominant amino acid in the system (Figure 3B). This finding aligns with literature that identifies proline as a major storage amino acid in cereals and as a crucial osmoprotectant during stress conditions (37). The sharp decline in proline content through successive fermentation stages suggests its utilization by fermentative microorganisms that often metabolize proline as an energy source to maintain redox balance driven by acidification and fermentation. This contributes to the development of characteristic sourdough-like flavors (38). During fermentation, proline also plays a protective function by stabilizing protein structures under stress conditions such as changes in pH and temperature (39). This explains its consistent appearance as a key discriminant metabolite differentiating malted sorghum inoculum from the final fermented maize product. Amino acids such as Aspartic Acid, Valine, Threonine, and Tyrosine demonstrated a noticeable increase in fermented samples, especially in FWM and FYM. Aspartic acid showed a significant elevation in fermented stages, which may reflect its role in microbial energy metabolism and as a precursor for other essential amino acids (40). Similarly, Valine and Threonine, both essential amino acids, were present in low concentrations initially but increased during fermentation, as available microorganisms consume sugars and convert them to other substrates like lactic acid, which in turn supported valine synthesis (41).

The IS displayed high levels of Alanine, Threonine, and Valine, in addition to considerable amounts of Proline, confirming its role as a source of free amino acids and enzymatic activity. The inclusion of malted sorghum not only contributes to starch hydrolysis but also enriches the amino acid pool, thereby supporting microbial activity and improving the sensory and nutritional profile of the beverage. The reduction in proline and the increase in essential amino acids such as Valine and Threonine improve the amino acid score of the beverage, enhancing its dietary protein quality. Furthermore, the liberation of amino acids during fermentation provides precursors for flavor compounds through Maillard and Strecker reactions, contributing to the characteristic taste of *amahewu*. This process may also facilitate the generation of bioactive peptides with antioxidant and antihypertensive properties, further positioning *amahewu* as a functional fermented cereal beverage.

### 3.4 Metabolite-metabolite correlation and pathway analysis.

Through correlation and pathway analyses, the identifiable metabolite markers can be linked to specific metabolic interactions and pathways, thereby offering mechanistic insights into the processes underlying the observed differences across production stages. Notably, metabolite–metabolite correlation analysis reveals the strength and direction of interactions, with positive correlations indicating coordinated increases or decreases, and negative correlations reflecting inverse concentration trends (42).

**Figure 4:**
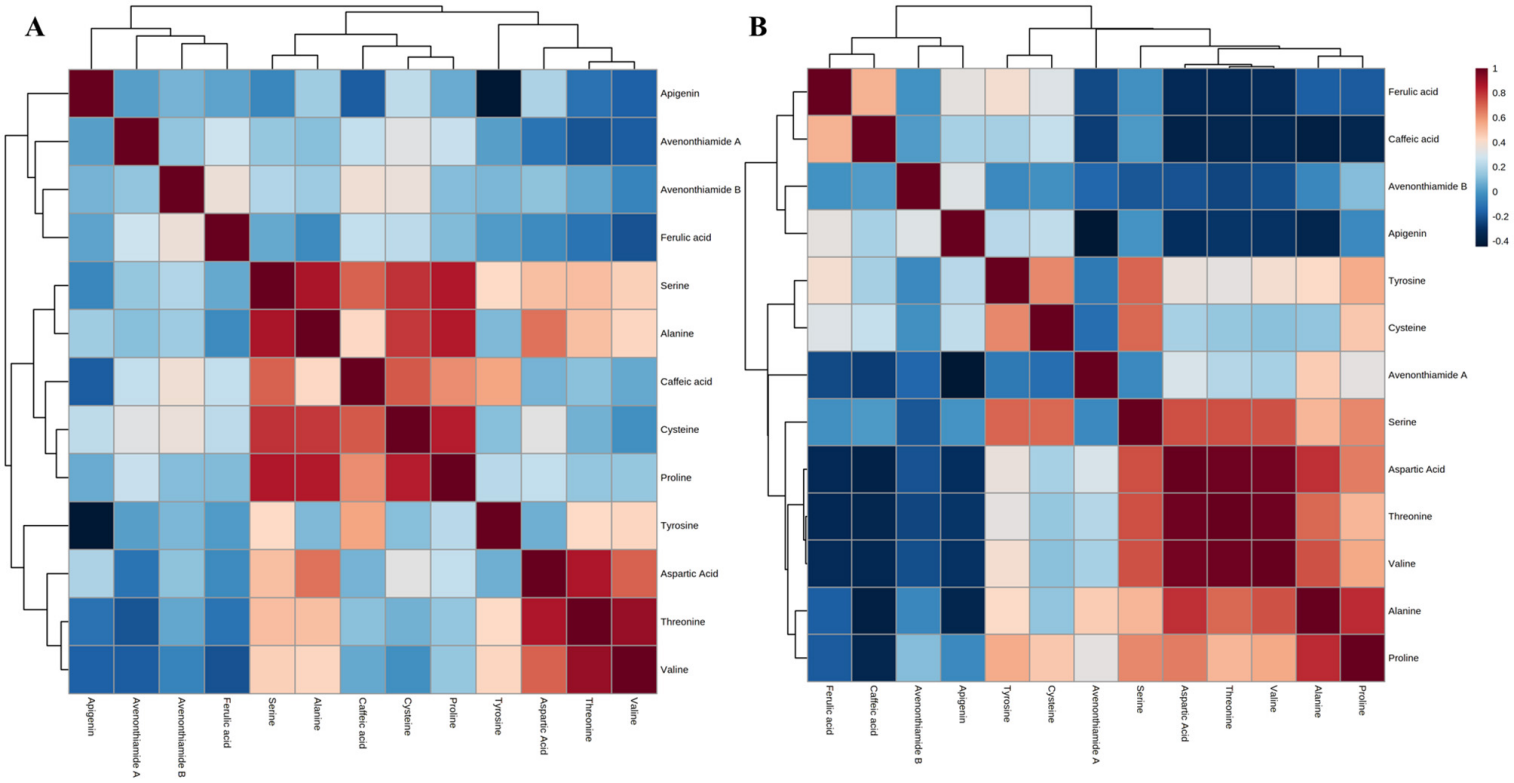
Metabolite-metabolite correlation analysis heatmaps between **(A)** white and **(B)** yellow maize production.

Strong positive correlations were observed between amino acids, namely serine, alanine, cysteine, proline, aspartic acid, threonine and valine and one phenolic compound as caffeic acid in white maize production (Figure 4A), whereas in yellow maize production, only amino acids had a strong positive correlation, namely proline, alanine, valine, threonine, aspartic acid and serine (Figure 4B). Correlation analysis proposes feed-forward and feed-back interaction of metabolites involved in the metabolic pathway. Amino acids in both productions exhibit a feed-forward interaction, where the presence of one amino acid promotes the production or accumulation of others, while phenolic compounds display a feedback interaction, resulting from the degradation of phenolic classes into derivatives that are often more bioactive than the parent compound (43).

**Figure 5:**
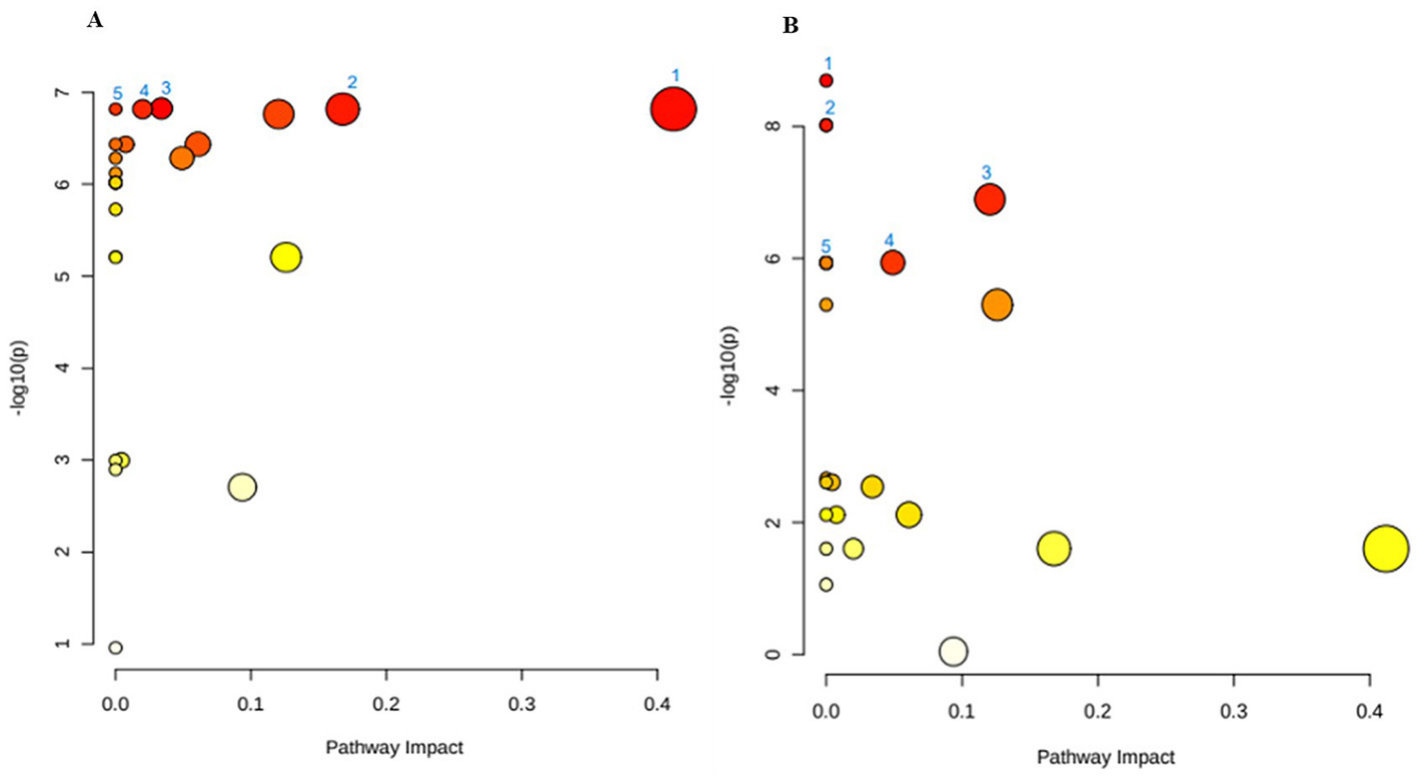
Metabolic pathway analysis of **(A)** white maize and **(B)** yellow maize production, with numbers (1–50) representing the main pathways and differential-difference of red = high and yellow = low. The graphs contain all the matched pathways (the metabolome) arranged by *p*-values (pathway enrichment analysis) on the y-axis, and the pathway impact values on the x-axis, further highlighted in Table 3 with the statistical *p*-values adjusted for multiple testing to account for numerous metabolic pathways that were all tested at the same time.

To map these feedback interactions of metabolites in the biological pathways, the functional analysis revealed impacted pathways (Figure 5, Table 3). The leading pathway in white maize production is isoquinoline alkaloid biosynthesis, a pathway that begins with the conversion of tyrosine and phenylalanine; isoquinoline alkaloids form a family of phytochemicals or secondary metabolites that are found in several plants' precursors (44). While isoquinoline alkaloids are not typically considered primary nutrients, their presence or altered production during fermentation can lead to modification of bioactive compounds in the final product. These compounds are known for their pharmacological properties, including anti-inflammatory and antioxidant activities (45). Therefore, the presence of isoquinoline alkaloids might interact synergistically with other nutritional elements in *amahewu*, such as vitamins, minerals, or polyphenols, potentially enhancing the overall properties of the food product. This is evident when the addition of *bambara* flour protein source enhances the nutritional value of *amahewu* by improving important AA lysine content and minerals, as well as an improvement in the amino profile (46). Tyrosine metabolism is the second leading metabolic pathway, which is significant as an upstream precursor for synthesizing a series of valuable natural products, such as phenolic acids (47, 48). The fermentation process may lead to tyrosine breakdown into bioactive metabolites like p-hydroxy phenylacetate and homovanillic acid, which could increase the functional value of *amahewu*. Also, changes in tyrosine metabolism during the fermentation process could alter the concentration of free tyrosine in *amahewu*, affecting its amino acid profile (47).

**Table 3:** Significant metabolic pathway altered in white and yellow maize production of *amahewu* from Metabolic Pathway Analysis (*MetPA*).

**Metabolic pathway**	**Total**	**Hits**	**Raw *p***	**–log10(p)**	**Holm adjust**	**FDR**	**Impact**
**White maize production**							
Phenylpropanoid biosynthesis	35	2	1.50 × 10^−7^	6.82	3.89 × 10^−6^	7.48 × 10^−7^	0.03
Isoquinoline alkaloid biosynthesis	6	1	1.52 × 10^−7^	6.82	3.89 × 10^−6^	7.48 × 10^−7^	0.41
Tyrosine metabolism	18	1	1.52 × 10^−7^	6.82	3.89 × 10^−6^	7.48 × 10^−7^	0.17
Phenylalanine, tyrosine and tryptophan biosynthesis	22	1	1.52 × 10^−7^	6.82	3.89 × 10^−6^	7.48 × 10^−7^	0.02
Glycine, serine and threonine metabolism	33	2	1.73 × 10^−7^	6.76	3.89 × 10^−6^	7.48 × 10^−7^	0.12
Sulfur metabolism	15	1	3.68 × 10^−7^	6.43	7.36 × 10^−6^	1.06 × 10^−6^	0.06
Glutathione metabolism	27	1	3.68 × 10^−7^	6.43	7.36 × 10^−6^	1.06 × 10^−6^	0.01
Cysteine and methionine metabolism	46	2	5.20 × 10^−7^	6.28	8.84 × 10^−6^	1.23 × 10^−6^	0.05
**Yellow maize production**							
Glycine, serine and threonine metabolism	33	2	1.28 × 10^−7^	6.89	2.94 × 10^−7^	8.31 × 10^−7^	0.12
Cysteine and methionine metabolism	46	2	1.16 × 10^−6^	5.94	2.55 × 10^−5^	2.76 × 10^−6^	0.05
Alanine, aspartate and glutamate metabolism	22	2	5.04 × 10^−6^	5.30	7.57 × 10^−5^	1.01 × 10^−5^	0.13
Sulfur metabolism	15	1	7.60 × 10^−3^	2.12	6.84 × 10^−2^	9.88 × 10^−3^	0.06
Phenylalanine, tyrosine and tryptophan biosynthesis	22	1	2.48 × 10^−2^	1.60	1.49 × 10^−1^	2.69 × 10^−2^	0.02

FDR, false discovery rate.

The findings from the production of *amahewu* using yellow maize and malted sorghum as inoculums indicate that amino acid metabolism was dominant. The metabolism of alanine, aspartate and glutamate, as well as glycine, serine and threonine (Gly-Ser-Thr), are the two leading impacted metabolic pathways. First, the amino acids were generated through enzymatic reactions with the help of present microorganisms, and then they were metabolized by the presumption of fermentation time, temperature and the application of inoculum. Alanine and aspartate can serve as substrates and precursors which are ultimately responsible for energy production and removing excess nitrogen from the body, while glutamate is widely known for its role as a flavor enhancer, contributing to the umami taste in foods (49). Similarly during the fermentation of *amahewu* from yellow maize, glutamate levels can increase, enhancing the sensory characteristics of the final product and leading to a more savory and appealing taste, making *amahewu* more enjoyable to consume. Additionally, it is speculated that antioxidants are produced through the Gly-Ser-Thr metabolic axis (50). Both Gly and Thr are critical components of glutathione, and since the considered factors in this study lead to their increase, *amahewu* could potentially have enhanced antioxidant properties, helping to combat oxidative stress and cellular damage.

## 4 Conclusion

This study revealed distinct metabolomic variations across processing stages and maize types during *amahewu* production, demonstrating that cooking, inoculation, and fermentation markedly reshape the beverage's biochemical composition. An increase in amino acids and phenolic content contributes enhanced the nutritional value of the beverage. The trend was consistent, throughout the study, as white maize showed steady metabolite increases, whereas yellow maize exhibited fluctuations of decreasing metabolites and lower fermentation efficiency. Thus, white maize–based *amahewu* showed more consistent and extensive metabolic remodeling than the yellow maize variant, reflecting higher fermentation efficiency, nutrient enrichment, and biochemical stability. Key metabolites such as valine, alanine, and apigenin emerged as reliable markers of fermentation progress. The chemometric analyses (PCA and OPLS-DA) confirmed clear separations among production stages, emphasizing the effect of fermentation on maize metabolism. Metabolite–metabolite correlation analysis revealed strong positive associations between amino acids and feedback-driven phenolic conversions. Pathway enrichment analysis identified isoquinoline alkaloid biosynthesis (in white maize) and alanine/aspartate/glutamate metabolism (in yellow maize) as central pathways shaping the metabolic outcomes. With further research, this approach could lead to improvements in the *amahewu* production process, ultimately resulting in a higher quality and more consistent final product.

## Data Availability

The raw data supporting the conclusions of this article will be made available by the authors, without undue reservation.
