# Supplementary material for: Targeted metabolomics to quantitatively profile changes in amino acids and phenolics at every step of amahewu production from two Zea Mays L. maize types (white and yellow)
Source: Front Nutr. 2026 Jan 12;12:1697672. doi: 10.3389/fnut.2025.1697672 (PMC12834512; doi:10.3389/fnut.2025.1697672)
Supplement: Supplementary file 1 [file Supplementary_file_1.pdf]

1.1 OPLS-DA models of data from white maize and yellow maize production stages comparisons.

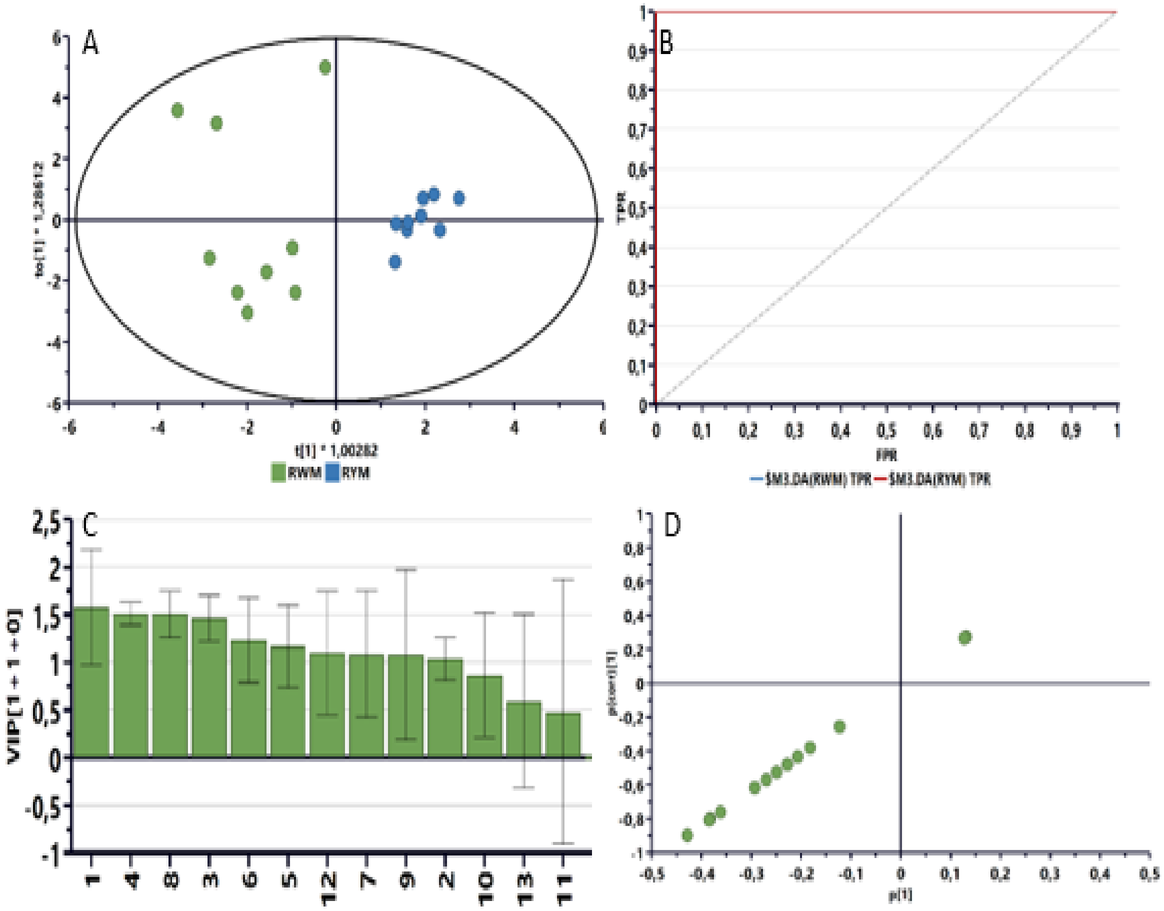

Figure S1: OPLS-DA modelling of RWM vs RYM: (**A**) OPLS-DA score plot separating RWM and RYM, (**B**) An ROC curve, (**C**) VIP plot and (**D**) S-plot loading. All computed under same model component 1+1+0 with R2X = 69.3%, R2Y = 85.5%, Q2 = 68.6.3% and CV-ANOVA, *p*-value of 2.93×10^{2}. RWM; raw white maize, RYM; raw yellow maize.

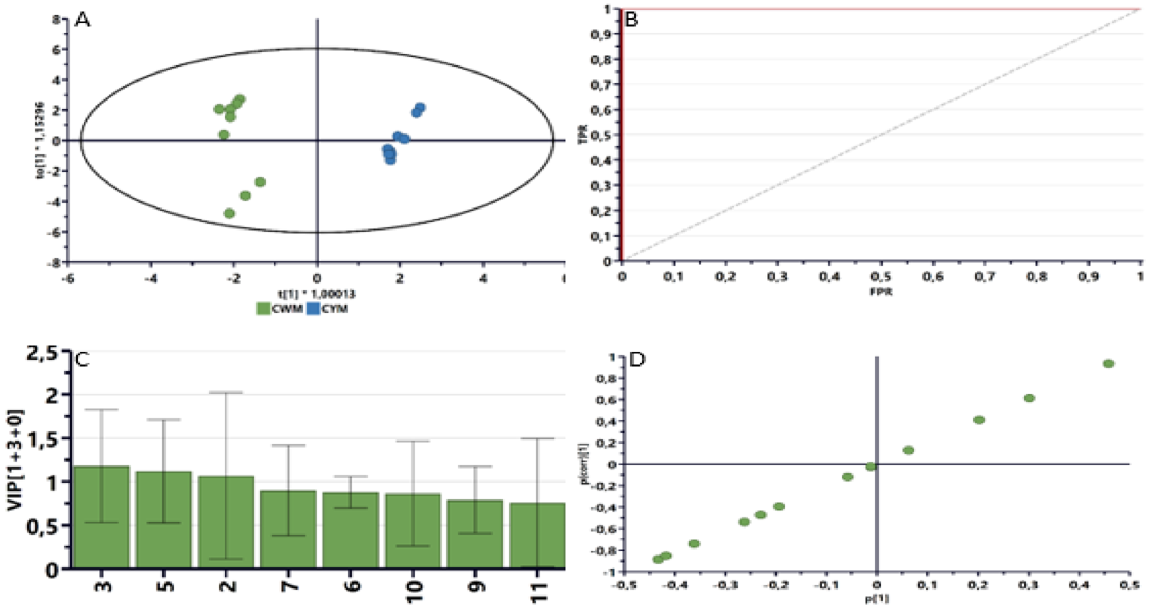

Figure S2: OPLS-DA modelling of CWM vs CYM: **(A)** OPLS-DA score plot separating CWM and CYM, (**B**) An ROC curve, (**C**) VIP plot and (**D**) S-plot loading. All computed under same model component 1+3+0 with R2X = 88.9%, R2Y = 98.0%, Q2 = 93.8% and CV-ANOVA, *p*-value of $3{\times}10^{-4}$. CWM = cooked white maize and CYM = cooked yellow maize**.**

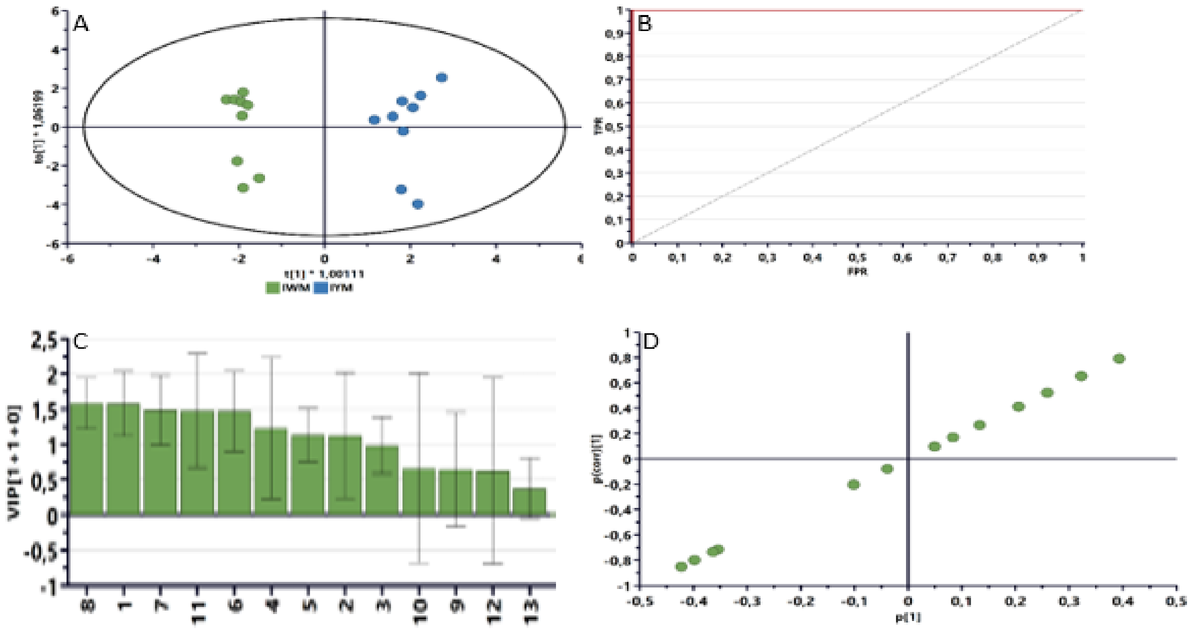

Figure S3: OPLS-DA modelling of IWM vs IYM: (**A**) OPLS-DA score plot separating IWM and IYM **(B)** An ROC curve**,** (**C**) VIP plot and (**D**) S-plot loading**.** All computed under same model component 1+1+0 with R2X = 62.5%, R2Y = 97.2%, Q2 = 93.9% and CV-ANOVA, *p*-value of 0. IWM = inoculated white maize and IYM = inoculated yellow maize.

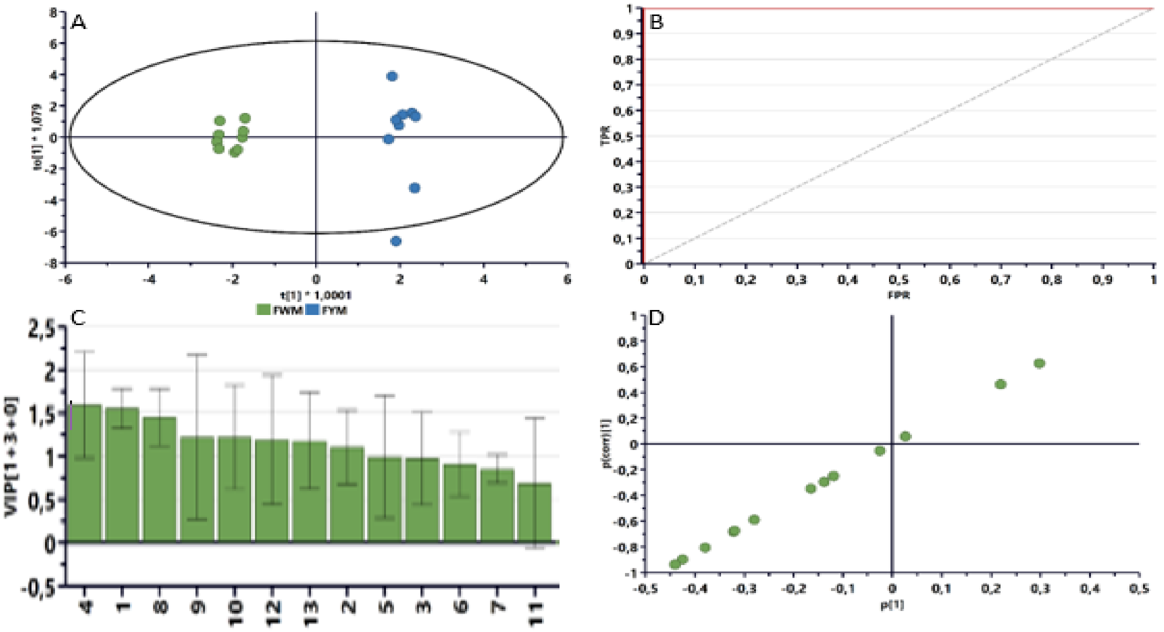

Figure S4: OPLS-DA modelling of FWM vs FYM: (**A**) OPLS-DA score plot separating FWM and FYM, (**B**) An ROC curve, (**C**) VIP plot and (**D**) S-plot loading. All computed under same model component 1+3+0 with R2X = 83.5%, R2Y = 98.5%, Q2 = 94.3% and CV-ANOVA, *p*-value of 2×10^{-4}. FWM = final white maize and FYM = final yellow maize.

1.2 OPLS-DA models of data from white maize samples.

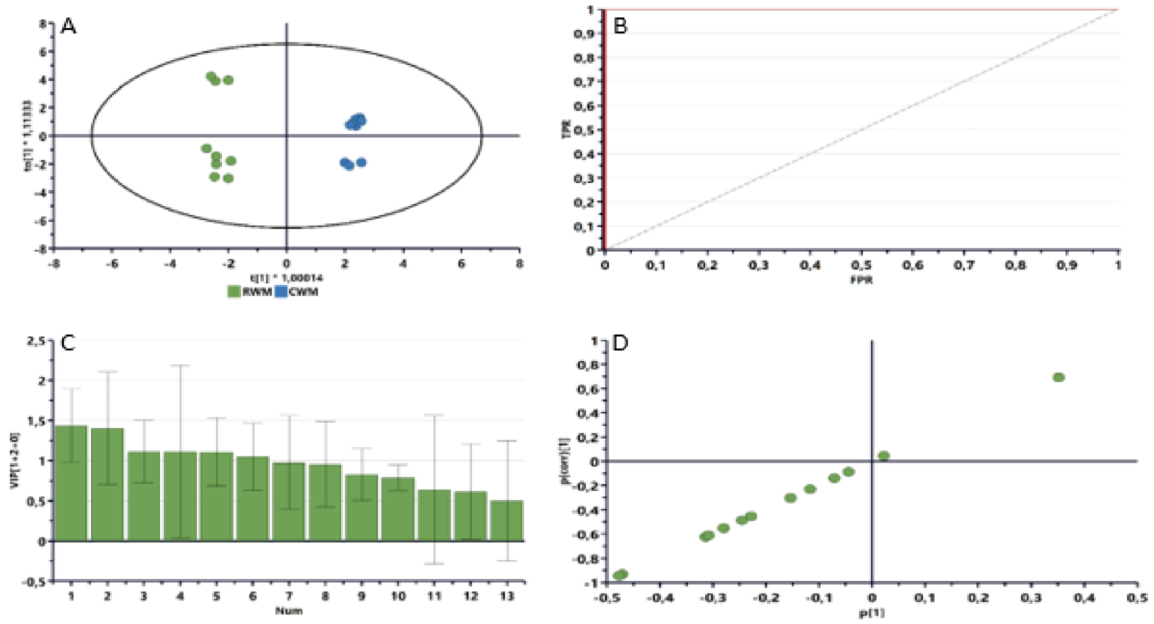

Figure S5: OPLS-DA modelling of RWM vs CWM: (**A**) OPLS-DA score plot separating RWM and CWM, (**B**) An ROC curve, (**C**) VIP plot and (**D**) S-plot loading. All computed under the same model component 1+2+0 with R2X = 82.8%, R2Y = 98.9%, Q2 = 97.2% and CV-ANOVA, *p*-value of 0. RWM = raw white maize and CWM = cooked white maize.

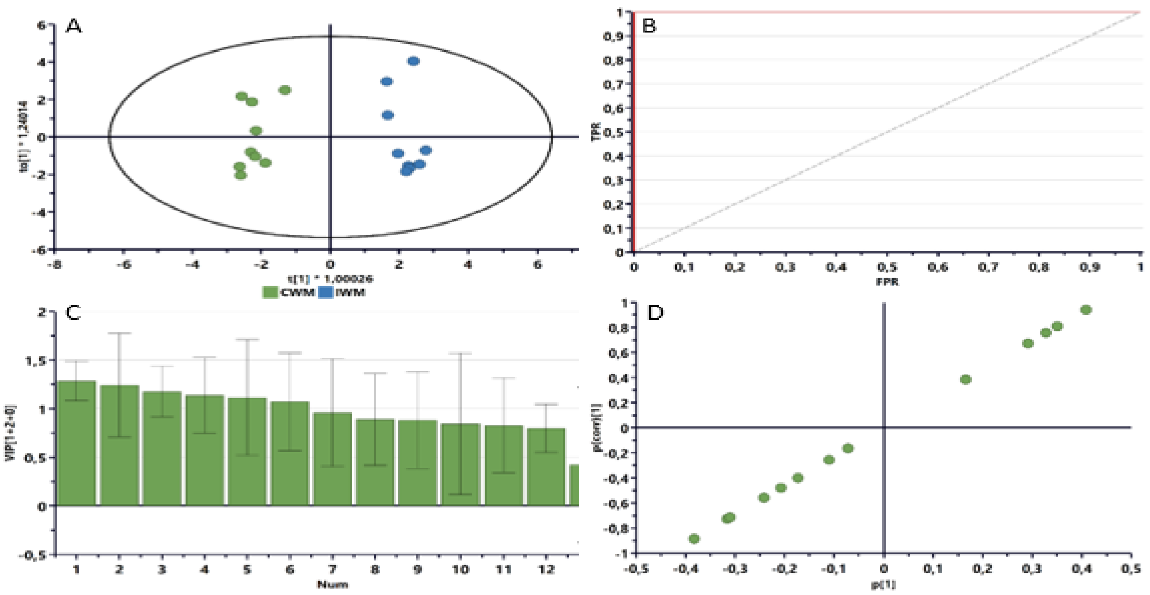

Figure S6: OPLS-DA modelling of CWM vs IWM: (**A**) OPLS-DA score plot separating CWM and IWM, (**B**) An ROC curve, (**C**) VIP plot and (**D**) S-plot loading. All computed under the same model component 1+1+0 with R2X = 74.8%, R2Y = 98.2%, Q2 = 97.3% and CV-ANOVA, *p*-value of 0. CWM = cooked white maize and IWM = inoculated white maize.

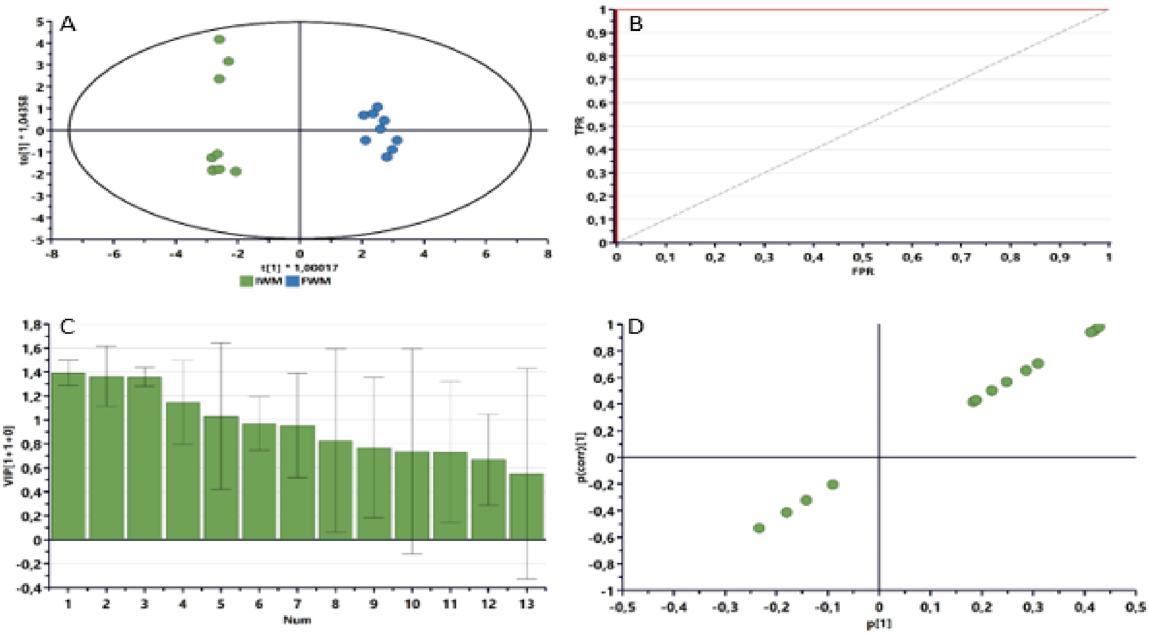

Figure S7: OPLS-DA modelling of IWM vs FWM: (**A**) OPLS-DA score plot separating IWM and FWM, (**B**) A ROC curve, (**C**) VIP plot and (**D**) S-plot loading. All computed under the same model component 1+1+0 with R2X = 67.2%, R2Y = 98.9%, Q2 = 97.7% and CV-ANOVA, *p*-value of 0. IWM = inoculated white maize and FWM = final white maize.

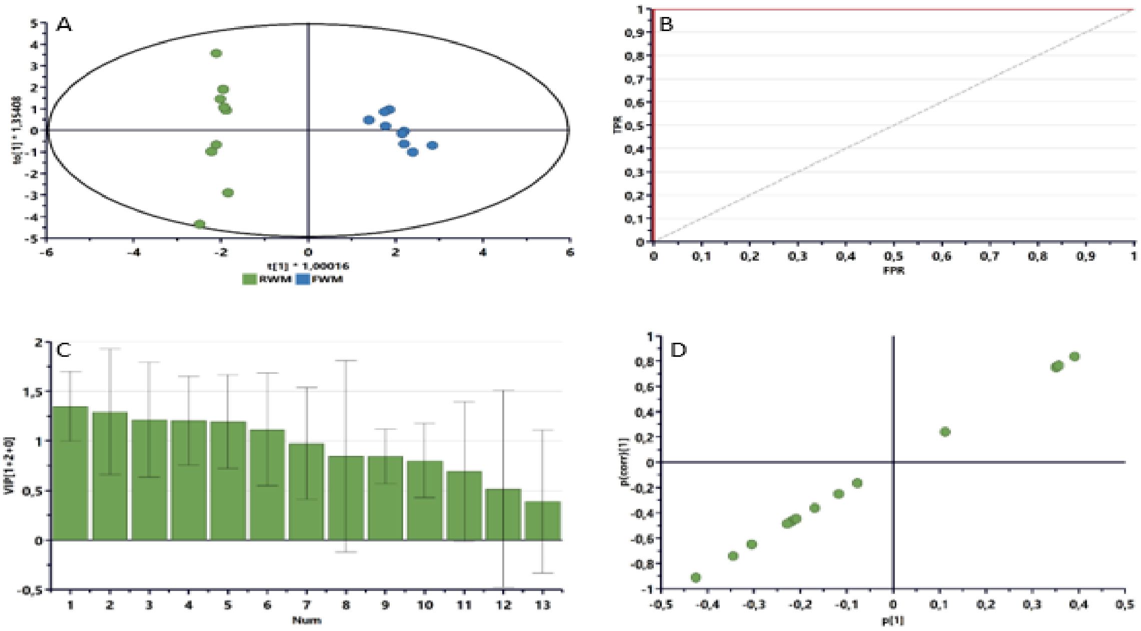

Figure S8: OPLS-DA modelling of RWM vs FWM: (**A**) OPLS-DA score plot separating RWM and FWM, (**B**) A ROC curve, (**C**) VIP plot and (**D**) S-plot loading. All computed under the same model component 1+2+0 with R2X = 55.2%, R2Y = 98.9%, Q2 = 95.1% and CV-ANOVA, *p*-value of 0. RWM = raw white maize and FWM = final white maize.

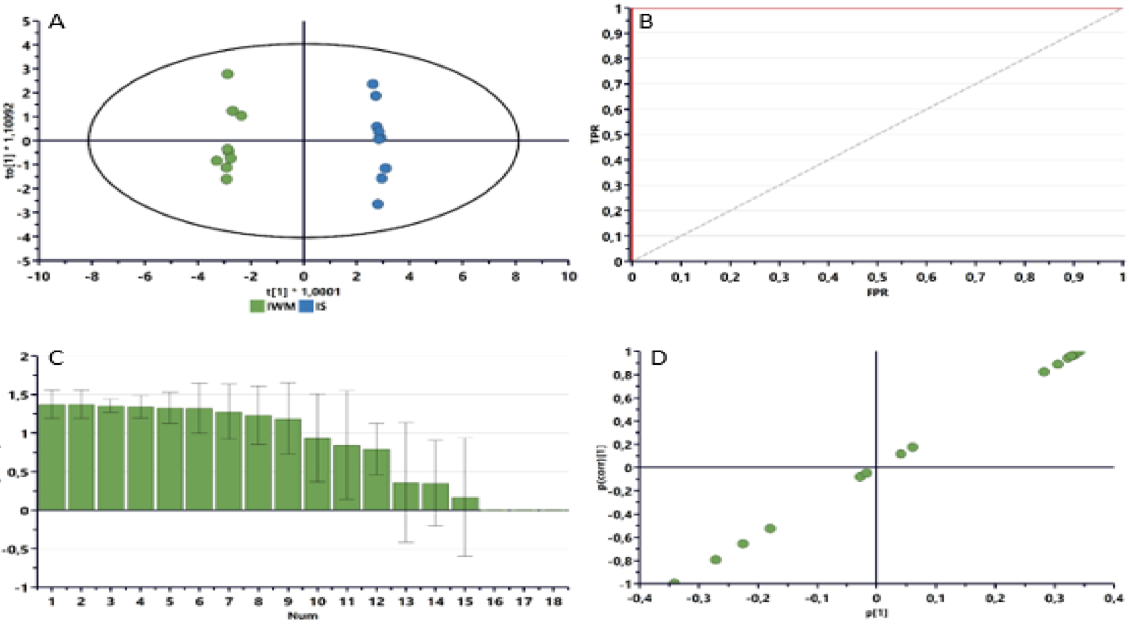

Figure S9: OPLS-DA modelling of IWM vs IS: (A) OPLS-DA score plot separating IWM and IS, (B) A ROC curve, (C) VIP plot and (D) S-plot loading. All computed under the same model component 1+3+0 with R2X = 69.1%, R2Y = 99.6%, Q2 = 99.1% and CV-ANOVA, *p*-value of 0.IWM = inoculated white maize and IS = malted sorghum.

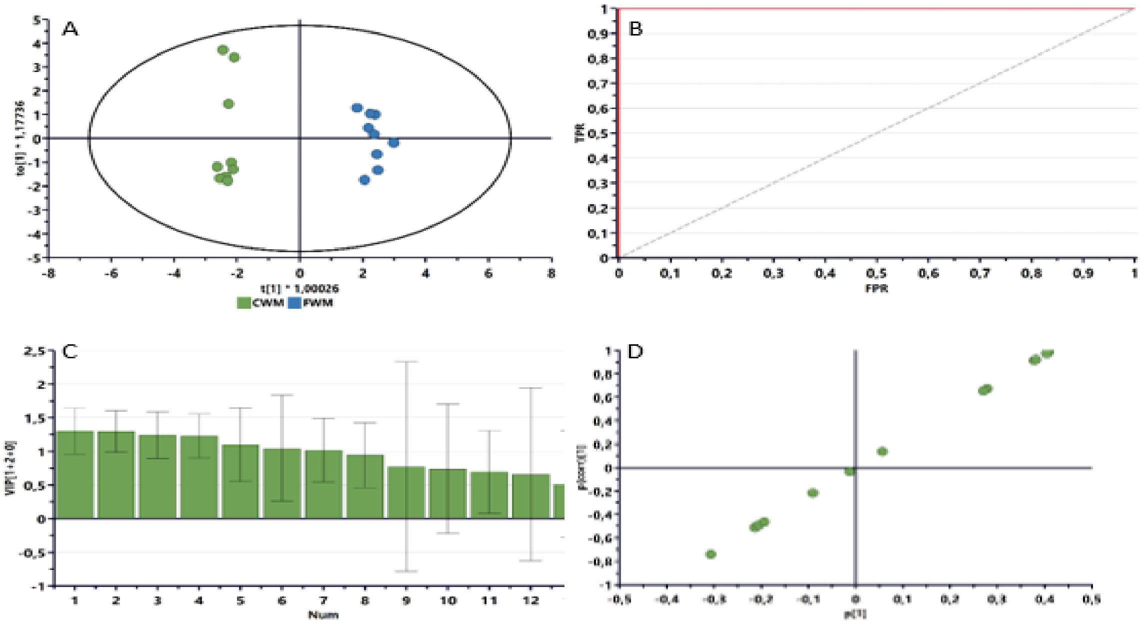

Figure S10: OPLS-DA modelling of CWM vs FWM: (**A**) OPLS-DA score plot separating CWM and FWM, (**B**) A ROC curve, (**C**) VIP plot and (**D**) S-plot loading. All computed under the same model component 1+1+0 with R2X = 68.4%, R2Y = 99.1%, Q2 = 98.1% and CV-ANOVA, *p*-value of 0.CWM = cooked white maize and FWM = final white maize.

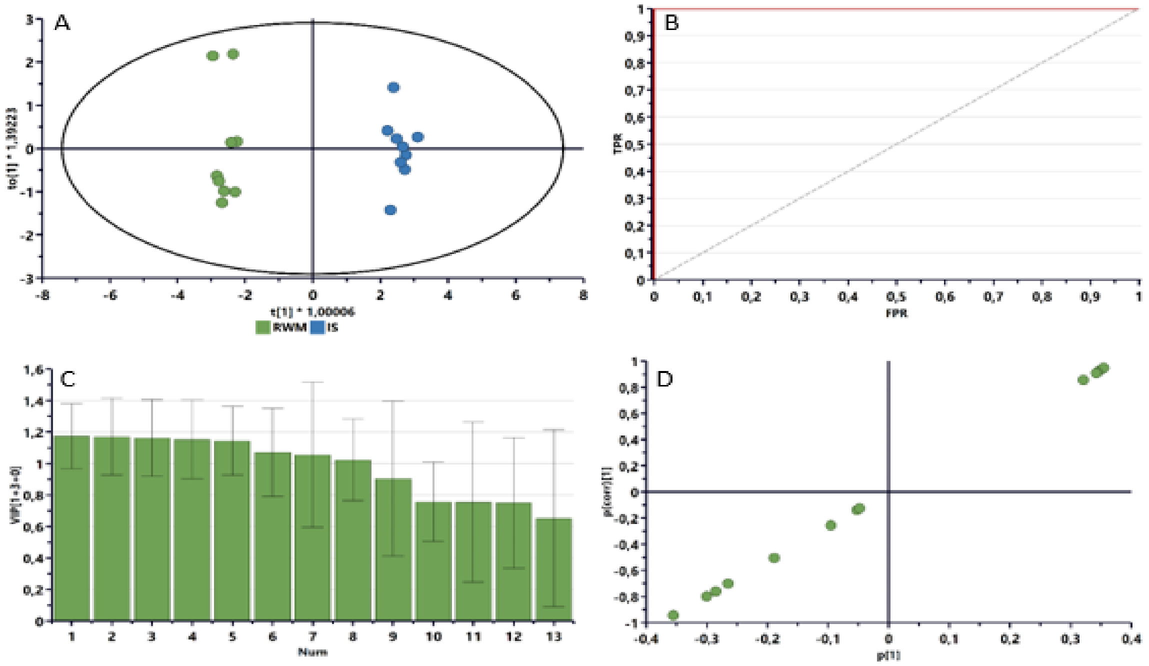

Figure S11: OPLS-DA modelling of RWM vs IS: (**A**) OPLS-DA score plot separating RWM and IS, (**B**) A ROC curve, (**C**) VIP plot and (**D**) S-plot loading. All computed under the same model component 1+1+0 with R2X = 73.3%, R2Y = 98.8%, Q2 = 97.3% and CV-ANOVA, *p*-value of 0.RWM = raw white maize and IS = malted sorghum.

*1.3 OPLS-DA models of data from yellow maize samples***.**

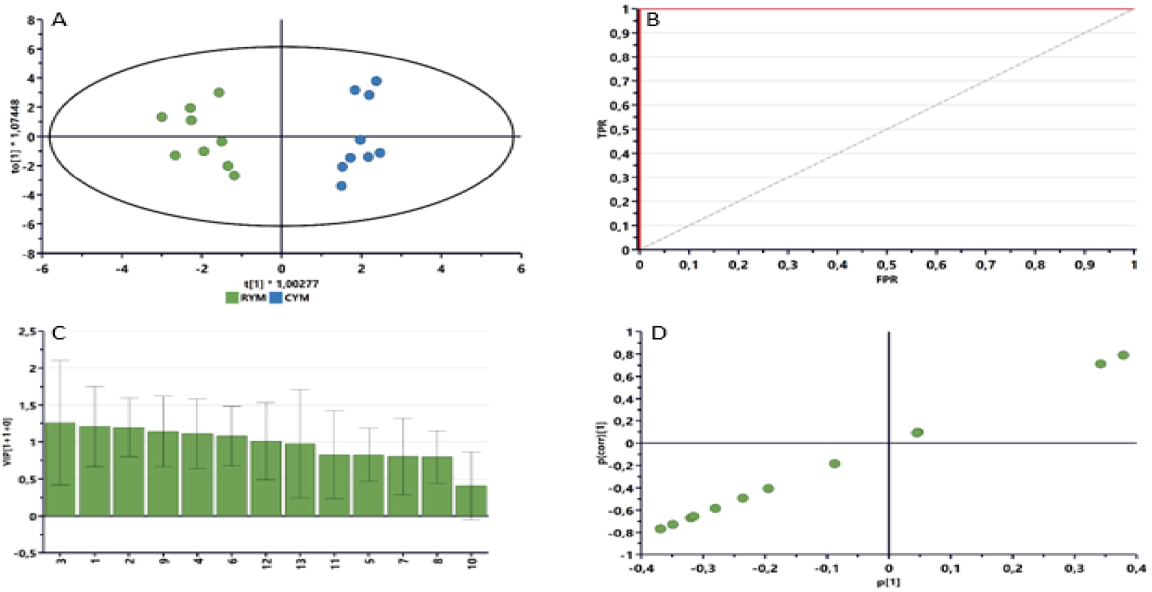

Figure S12: OPLS-DA modelling of RYM vs CYM: (**A**) OPLS-DA score plot separating RYM and CYM, (**B**) A ROC curve, (**C**) VIP plot and (**D**) S-plot loading. All computed under the same model component 1+2+0 with R2X = 81.6%, R2Y = 98.4%, Q2 = 96.3% and CV-ANOVA, *p*-value of 0. RYM = raw yellow maize and CYM = cooked yellow maize.

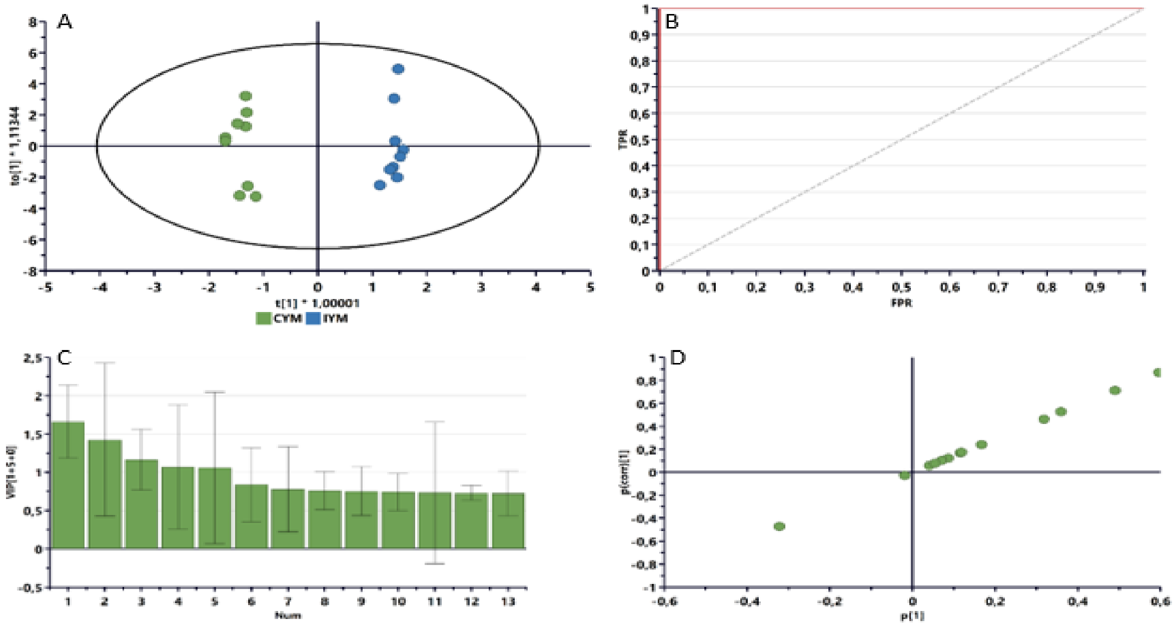

Figure S13: OPLS-DA modelling of CYM vs IYM: (**A**) OPLS-DA score plot separating CYM and IYM, (**B**) A ROC curve, (**C**) VIP plot and (**D**) S-plot loading. All computed under the same model component 1+3+0 with R2X = 86.2%, R2Y = 99.6%, Q2 = 98.6% and CV-ANOVA, *p*-value of 0. CYM = cooked yellow maize and IYM = inoculated yellow maize.

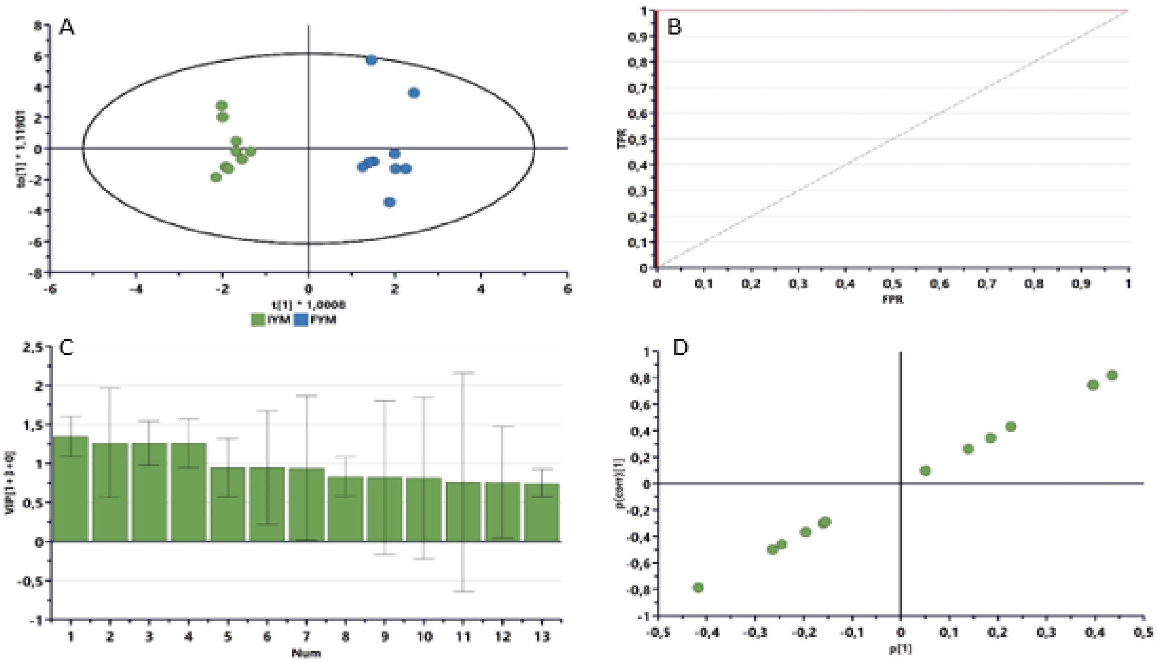

Figure S14: OPLS-DA modelling of IYM vs FYM: (**A**) OPLS-DA score plot separating IYM and FYM, (**B**) A ROC curve, (**C**) VIP plot and (**D**) S-plot loading. All computed under the same model component 1+2+0 with R2X = 76.5%, R2Y = 98.9%, Q2 = 97.4% and CV-ANOVA, *p*-value of 0. IYM = inoculated yellow maize and FYM = final yellow maize.

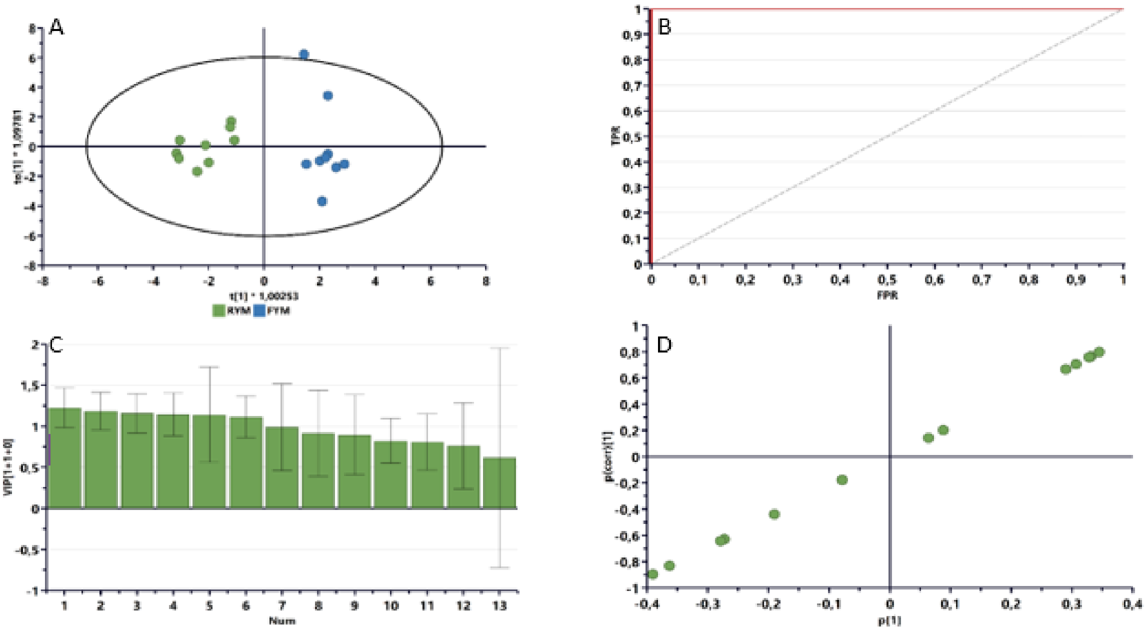

Figure S15: OPLS-DA modelling of RYM vs FYM: (**A**) OPLS-DA score plot separating RYM and FYM, (**B**) A ROC curve, (**C**) VIP plot and (**D**) S-plot loading. All computed under the same model component 1+2+0 with R2X = 83.4%, R2Y = 98.8%, Q2 = 96.5% and CV-ANOVA, *p*-value of 0. RYM = raw yellow maize and FYM = final yellow maize.

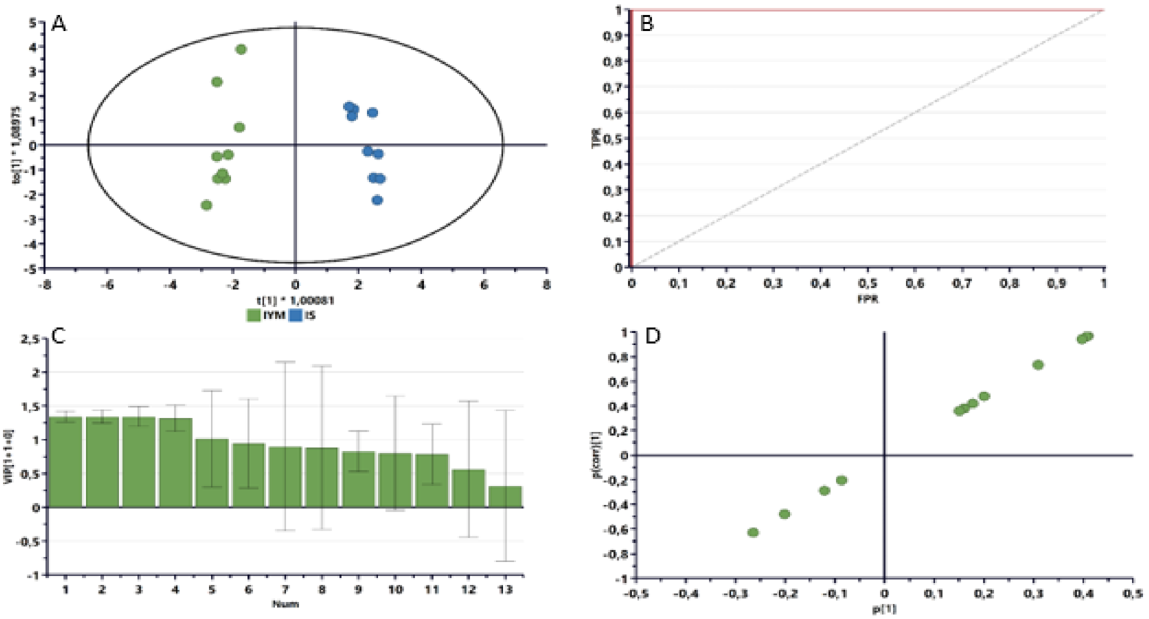

Figure S16: OPLS-DA modelling of IYM vs IS: (**A**) OPLS-DA score plot separating IYM and IS, (**B**) A ROC curve, (**C**) VIP plot and (**D**) S-plot loading. All computed under the same model component 1+1+0 with R2X = 67.6%, R2Y = 98.9%, Q2 = 97.4% and CV-ANOVA, *p*-value of 0. IYM = inoculated yellow maize and IS = malted sorghum.

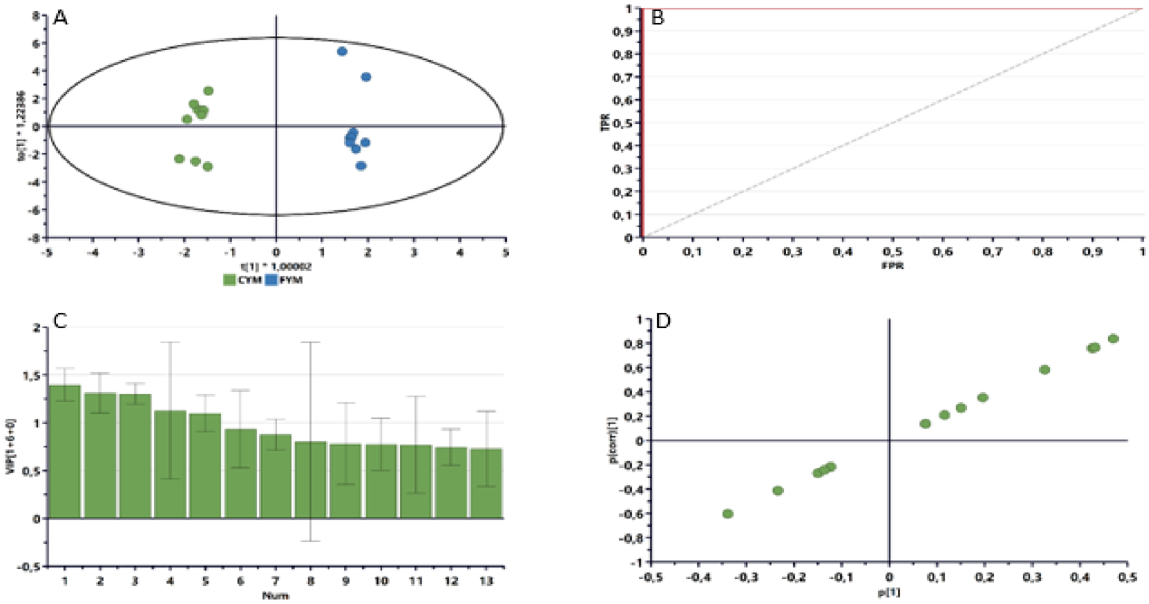

Figure S17: OPLS-DA modelling of CYM vs FYM**:** (**A**) OPLS-DA score plot separating CYM and FYM, (**B**) A ROC curve, (**C**) VIP plot and (**D**) S-plot loading. All computed under the same model component 1+3+0 with R2X = 87.2%, R2Y = 99.7%, Q2 = 98.6% and CV-ANOVA, *p*-value of 0. CYM = cooked yellow maize and FYM = final yellow maize.

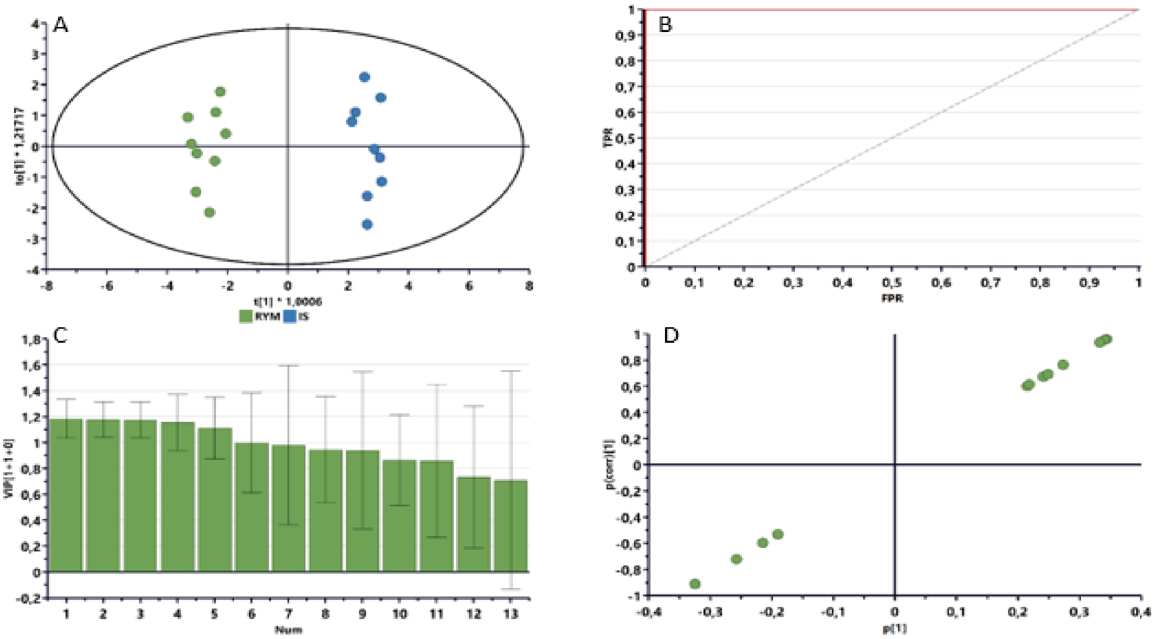

Figure S18: OPLS-DA modelling of RYM vs IS**:** (**A**) OPLS-DA score plot separating RYM vs IS, (**B**) A ROC curve, (**C**) VIP plot and (**D**) S-plot loading. All computed under the same model component 1+1+0 with R2X = 76.4%, R2Y = 98.9%, Q2 = 98.3% and CV-ANOVA, *p*-value of 0. RYM = raw yellow maize and IS = malted sorghum.
